# Unveiling the therapeutic role of Dachaihu decoction in acute cholecystitis: a comprehensive systematic review and meta-analysis of its efficacy and safety

**DOI:** 10.3389/fphar.2024.1497072

**Published:** 2024-11-27

**Authors:** Xin-xin Liu, Ying-qi Ma, Ling-yao Kong, You-zhu Su, Nicola Robinson, Jian-ping Liu

**Affiliations:** ^1^ Centre for Evidence-Based Chinese Medicine, Beijing University of Chinese Medicine, Beijing, China; ^2^ The First Clinical Medical College, Shandong University of Traditional Chinese Medicine, Jinan, Shandong, China; ^3^ School of Health and Social Care, London South Bank University, London, United Kingdom; ^4^ Department of Community Medicine, The National Research Center in Complementary and Alternative Medicine (NAFKAM), Faculty of Health Science, UiT The Arctic University of Norway, Tromsø, Norway

**Keywords:** Dachaihu decoction, acute cholecystitis, clinical efficacy, systematic review, meta-analysis

## Abstract

**Background:**

Dachaihu decoction (Dachaihu tang) plays a crucial role in treating acute illnesses. Recently, a significant number of clinical studies on Dachaihu decoction for acute cholecystitis (AC) have been published. This study was conducted to assess the efficacy and safety of Dachaihu decoction in patients with this condition.

**Methods:**

To identify relevant randomized controlled trials (RCTs), eight databases and three clinical trial registries were searched from inception to 30 June 2024. Two researchers independently screened and extracted data from eligible studies using EndNote X9 and Microsoft Office Excel 2019. RoB 2.0 was used to assess the risk of bias in the included studies. Stata 17.0 was used for data analysis. Publication bias and its impact on result stability were evaluated using a funnel plot and the “trim-and-fill” method. The quality of evidence was graded using the GRADE assessment system.

**Results:**

Thirty-three RCTs involving 2,851 participants were included. The treatment group demonstrated improved clinical efficacy (RR = 1.18; 95% CI = 1.13 to 1.24), significantly reduced length of hospital stay (MD = −1.78 days; 95% CI = –2.02 to −1.53), and the incidence of adverse events (RR = 0.31; 95% CI = 0.20 to 0.48). Additionally, there appeared to be reductions in the time for abdominal pain to resolve (MD = −1.92 days; 95% CI = –2.33 to −1.51), fever to disappear (MD = −1.52 days; 95% CI = –1.90 to −1.14), white blood cell count to return to normal (MD = −2.89 days; 95% CI = –3.32 to −2.46), alanine aminotransferase (ALT) levels (MD = −11.88 U/L; 95% CI = –15.29 to −8.47), aspartate aminotransferase (AST) levels (MD = −8.74 U/L; 95% CI = –9.76 to −7.72), neutrophil percentage (MD = −9.68; 95% CI = –11.33 to −8.03), TNF-α levels (SMD = −2.10 pg/L; 95% CI = –2.43 to −2.78), and certainty of evidence (moderate-to-low certainty).

**Conclusion:**

Dachaihu decoction may be an effective botanical formula for managing AC and a lower incidence of adverse events. However, due to the substantial risk of bias and heterogeneity across the included studies, these findings should be interpreted with caution and require further validation through well-designed, high-quality trials.

**Systematic Review registration:**

https://www.crd.york.ac.uk/PROSPERO/display_record.php?RecordID=573332.

## 1 Introduction

Acute cholecystitis (AC) is an acute infectious gallbladder disease caused by cystic duct obstruction, chemical stimulation, and bacterial infection. It represents 10% of all acute abdominal cases in clinical practice (Abdulrahman et al., 2022; Kimura et al., 2007; China Association Of Integrative Medicine Emergency Medicine Committee et al., 2019). The clinical manifestations include paroxysmal colic in the right upper abdomen, often accompanied by nausea, vomiting, fever, and jaundice. The incidence of AC increases among individuals aged 50 and above, with an overall mortality rate of approximately 3%, increasing significantly in patients with comorbidities. In cases without gallstones, mortality can escalate to 15%–40% (Bedirli et al., 2001; González-Castillo et al., 2021). This higher risk is not only due to AC being secondary to critical illnesses—such as trauma, surgery, shock, burns, sepsis, total parenteral nutrition, and mechanical ventilation—but also because it is more likely to lead to gangrene, perforation, and pyothorax, or empyema compared to calculous cholecystitis (Kimura et al., 2007; Gluhovschi et al., 2023).

AC arises from a confluence of factors, including systemic inflammatory responses, multiorgan dysfunction, and gallbladder damage caused by gallstones and infection (Markaki et al., 2021; Nitzan et al., 2017; Regimbeau et al., 2014; Halpin, 2014). Treatment typically involves medication (such as anti-infection agents, antispasmodics, and analgesics) and surgical interventions. However, in elderly patients with comorbidities, a conservative approach is favored as it effectively relieves symptoms and controls infection during the acute phase (Mencarini et al., 2024). Despite targeting the initial pathological event, there are no specific agents for AC treatment. The risk of postoperative infectious complications in grade 1 and 2 AC is approximately 17% (Regimbeau et al., 2014). The role of antibiotics and surgical interventions, whether administered early in the disease or during the perioperative period, remains a topic of debate (Singh et al., 2023; Dietrich et al., 2024). Therefore, on the basis of modern medical treatments, exploring alternative treatments for AC is crucial.

A large number of traditional Chinese medicine (TCM) formulas are utilized in Chinese clinical practice to treat diseases with complex mechanisms. Recently, the focus has shifted toward combination therapies involving multi-target and multi-component drugs, which has become fundamental in exploring the molecular mechanisms of TCM prescription components for disease treatment (Li et al., 2023). TCM formulas have demonstrated significant efficacy and safety over thousands of years of clinical practice (Sheehan et al., 1992; Deng and Xu, 2017). Dachaihu decoction (Dachaihu Tang), a classic TCM formula, was documented in the Treatise on Febrile Diseases by Zhang Zhongjing, a prominent traditional Chinese physician from 150 to 219 AD. Comprising eight botanical drugs, namely, *Bupleurum chinense* DC*.* [Apiaceae; *Bupleuri radix*], *Scutellaria baicalensis* Georgi [Lamiaceae; *Scutellariae radix*], *Citrus aurantium* L*.* [Rutaceae; *Citri aurantii fructus immaturus*], *Pinellia ternata* (Thunb.) *Makino* [Araceae*; Pinelliae rhizoma*], *Rheum officinale* Baill. [Polygonaceae*; Rhei radix et rhizoma*], *Paeonia lactiflora* Pall. [Paeoniaceae*; Paeoniae radix alba*], *Zingiber officinale* Roscoe [Zingiberaceae*; Zingiberis rhizoma*], and *Ziziphus jujuba* Mill. [Rhamnaceae*; Ziziphi jujubae fructus*], it is noted for its ability to relieve abdominal pain, reduce fever, alleviate gastrointestinal symptoms, and lower inflammation (Bi et al., 2023).

In 1994, Japanese Kampo medicine scholars first demonstrated its capability to ameliorate liver injury in a mechanistic study of Dachaihu decoction (Ji et al., 2024). The main bioactive components include saikosaponin B2, baicalin, baicalein, epiberberine, naringin, hesperidin, neohesperidin, and nobiletin. These efficacy markers may influence various signaling pathways such as MAPK, NF-κB, and Toll-like receptors by targeting molecules like JUN, TNF, EGFR, MAPK3, RELA, TNF, HIF1A, AKT1, IGF1R, CREB1, and SIRT1, HIF-1, PI3K-AKT, and AMPK, thus regulating the ERK1/ERK2 cascade, NF-κB transcription factor activity, inflammatory responses, glucose metabolism, angiogenesis, and adipocyte differentiation (Mao et al., 2017; Ohta et al., 1995). Dachaihu decoction protects the liver, promotes bile flow, and exerts anti-inflammatory effects, making it a commonly prescribed botanical drug treatment for AC among clinicians (Bi et al., 2023; Zhou, 2020). It can reduce inflammatory responses and alleviate gastrointestinal symptoms in patients with AC by regulating CRP levels and inflammatory factors (TNF-α, IL-6, and IL-8), without apparent side effects (Liu et al., 2021). Given the global prevalence of AC, the high rate of perioperative infections, and the limitations of Western medications such as anti-infective drugs and analgesics in managing this condition, along with extensive clinical study data from clinical studies, there is a pressing need for a comprehensive review of existing research. This study aims to review the relevant literature to summarize previous findings on the efficacy of Dachaihu decoction alone or in combination with conventional treatment for managing AC, thereby providing a deeper understanding of its overall efficacy and safety in AC treatment.

## 2 Methods

This study has been registered on the PROSPERO platform (Registration No.: CRD42024573332; https://www.crd.york.ac.uk/PROSPERO/display_record.php?RecordID=573332).

### 2.1 Eligibility criteria

(1) Participants: Individuals aged 18 years or older who met the diagnostic criteria for AC, as outlined in the Tokyo Guidelines (2018) (Kimura et al., 2007), including ① obvious right upper-abdominal pain with tenderness or a positive Murphy sign; ② fever, elevated C-reactive protein, or elevated white blood cell count; and ③ imaging examination suggested acute cholecystitis. The diagnosis of acute cholecystitis could be confirmed if any one of the criteria in ①, ②, and ③ was met. Exclusion criteria encompassed patients with severe cardiovascular, hepatic, or renal insufficiency; malignant tumors or significant organic lesions; a history of severe neurological dysfunction or mental illness; and pregnant or lactating women, or those allergic to Dachaihu decoction.
(2) Interventions: The treatment group received Dachaihu decoction alone/or in combination with the conventional treatment (CT)/laparoscopic cholecystectomy (LC). The composition of Dachaihu decoction includes *B. chinense* DC. [Apiaceae; *B. radix]*, *S. baicalensis* Georgi [Lamiaceae; *S. radix*], *C. aurantium* L. [Rutaceae; *C. aurantii fructus immaturus*], *P. ternata* (Thunb.) Makino [Araceae; *P. rhizoma*], *R. officinale* Baill. [Polygonaceae; *R. radix et rhizoma*], *P. lactiflora* Pall. [Paeoniaceae; *P. radix alba*], *Z. officinale* Roscoe [Zingiberaceae; *Z. rhizoma*], and *Z. jujuba* Mill. [Rhamnaceae; *Z. jujubae fructus].* All botanical drugs were selected and reported according to the guidelines of the “Consortium for Phytochemical Characterization of Medicinal Plants (ConPhyMP)” (Heinrich et al., 2022; Heinrich et al., 2020). Moreover, in accordance with the modification principles of TCM, there were no restrictions on the dosage (grams), slight adjustments, formulation, or the route of administration for this decoction. Information on the botanical drugs, standard dosage (grams), and decoction preparation method of Dachaihu decoction is given in Table 1 and Supplementary Material S1.
(3) Comparisons: The control group was treated with CT, including antibiotics (β-lactams, 4-quinolones, and nitroimidazoles), along with antispasmodic, hepatoprotective, and choleretic drugs. Surgical intervention (LC), specifically laparoscopic cholecystectomy, was carried out when indicated.
(4) Outcomes: ① Clinical efficacy was assessed using criteria adapted from the Guiding Principles for Clinical Research of New Chinese Medicines (Ministry of Health of the PRC, 2002): cured—significant improvement in symptoms and signs, evidence point reduction ≥95%; obvious efficacy—notable improvement in symptoms and signs, evidence point reduction ≥70% but <95%; efficacy—some improvement in symptoms and signs, evidence point reduction ≥30% but <70%; inefficacy—no significant improvement in symptoms and signs, evidence point reduction <30%; calculated using the nimodipine method—efficacy index (%) = [(pre-treatment points - post-treatment points)/pre-treatment points × 100%]. ② Time for resolution of abdominal pain. ③ Time for resolution of fever. ④ Time for white blood cell counts to normalize. ⑤ Length of hospital stay. ⑥ Levels of alanine aminotransferase (ALT). ⑦ Levels of aspartate aminotransferase (AST). ⑧ Neutrophil percentage (NEUT%). ⑨ Levels of tumor necrosis factor-α (TNF-α). ⑩ Incidence of adverse events. Primary outcomes included ⑤and⑩, and secondary outcomes included ①, ②, ③, ④, ⑥, ⑦, ⑧, and ⑨.
(5) Study design: All randomized controlled trials (RCTs).

**TABLE 1:** Basic information on botanical drugs in Dachaihu decoction.

Common English name	Botanical Latin name	Authorities	Family	Parts and form used	Processing of botanical drugs	Dosage (grams)	Main bioactive compounds	Medicinal source (Pharmacopoeia)
Bupleuri radix	*Bupleurum chinense* DC.	Augustin Pyramus de Candolle	Apiaceae	Root and dried root	Remove impurities and remaining stems, wash thoroughly, moisten until fully softened, slice thickly, and dry	15	Saikosaponins, flavonoids, essential oils, and phytosterols	China Pharmacopoeia (2015)
Chinese skullcap root	*Scutellaria baicalensis* Georgi	Johann Gottlieb Georgi	Lamiaceae	Root and dried root	Remove impurities and boil in water for 10 min. Take out and let it fully soften, then slice thinly, and dry. Alternatively, steam for 30 min, then slice thinly, and dry (avoiding direct sunlight)	9	Baicalin, baicalein, wogonin, and wogonoside	U.S. Pharmacopoeia USP 43 (2020)
Citron fruit	*Citrus aurantium* L.	Carl Linnaeus	Rutaceae	Fruit and dried ripe fruit	Remove impurities, wash thoroughly, moisten until fully softened, slice thinly, and dry	9	Synephrine, naringin, hesperidin, and limonin	China Pharmacopoeia (2010)
Banxia	*Pinellia ternata* (Thunb.) Makino.	Carl Peter Thunberg	Araceae	Tuber and dried tuber	Separate *Pinellia ternata* by size and soak in water until fully softened. Remove and set aside. Boil *Glycyrrhiza uralensis* twice, combine the decoctions, and mix with lime solution. Add the soaked *Pinellia*, stir 1–2 times daily, keeping pH above 12. Soak until the cross section is uniformly yellow and slightly tingling to taste. Remove, wash, and dry	9	Pinellia alkaloids, pinellin, polysaccharides, and volatile oils	China Pharmacopoeia (2015)
Rhubarb	*Rheum officinale* Baill	Henri Ernest Baillon	Polygonaceae	Rhizome, root, and dried root and rhizome	Remove impurities, wash thoroughly, moisten until softened, cut into thick slices or chunks, and air-dry	6	Anthraquinones, rhein, chrysophanol, emodin-8-glucoside, and aloe emodin	European Pharmacopoeia, 7th edn. (2012)
Chinese peony	*Paeonia lactiflora* Pall	Peter Simon Pallas	Paeoniaceae	Root and dried root	Wash thoroughly, moisten until softened, slice thinly, and dry	9	Paeoniflorin, albiflorin, and catechins	U.S. Pharmacopoeia USP 39 (2016)
Ginger	*Zingiber officinale* Roscoe	William Roscoe	Zingiberaceae	Rhizome and dried rhizome	Remove impurities and wash thoroughly. Slice thickly before use	15	Gingerols, shogaols, zingerone, volatile oils, and flavonoids	U.S. Pharmacopoeia USP 43 (2020)
Ba	*Ziziphus jujuba* Mill	Philip Miller	Rhamnaceae	Fruit and dried ripe fruits	Remove impurities, wash thoroughly, and sun-dry. Break open or remove the seeds before use	9	Polysaccharides, saponins, and vitamin C	U.S. Pharmacopoeia USP 39 (2016)

Note: According to the Chinese Pharmacopoeia, the decoction preparation method for traditional Chinese medicine (TCM) is as follows: weigh *Bupleurum chinense* DC. [Apiaceae; *Bupleuri radix],* Scutellaria baicalensis *Georgi* [Lamiaceae; *Scutellariae radix], Citrus aurantium* L. [Rutaceae; *Citri aurantii fructus immaturus], Pinellia ternata (Thunb.)* Makino [Araceae; *Pinelliae rhizoma], Rheum officinale* Baill. [Polygonaceae; *Rhei radix et rhizoma], Paeonia lactiflora* Pall. [Paeoniaceae; *Paeoniae radix alba], Zingiber officinale* Roscoe [Zingiberaceae; *Zingiberis rhizoma]*, *and Ziziphus jujuba* Mill. [Rhamnaceae; *Ziziphi jujubae fructus]*, according to the specified dosages (grams). Remove any impurities and wash thoroughly. Place the botanical drugs in a clean container, add sufficient water, and soak for approximately 30 min until softened. Transfer both the botanical drugs and soaking liquid to a clay or stainless-steel pot, add 6–10 times the herb weight in water, bring to a boil, and then simmer for 30 min. After the initial decoction, strain the liquid, add more water to the residue, and decoct again for 20–25 min. Combine and mix the two decoctions and then filter through a fine sieve or cloth for clarity. Pour the decoction into a clean container while hot, seal tightly, and consume within 24 h. For longer storage, reboil to sterilize and refrigerate. It should be taken warm, in divided doses, with dosage and frequency adjusted based on medical advice.

### 2.2 Literature search

Two researchers (Xin-xin Liu and You-zhu Su) systematically searched eight databases, i.e., four English databases (Web of Science, Cochrane Database of Systematic Reviews, PubMed, and EMBASE) and four Chinese databases (CNKI, Wanfang, VIP, and SinoMed). Additionally, three clinical trial registration platforms (International Clinical Trials Registry Platform, ClinicalTrials.gov, and Chinese Clinical Trial Registry) were searched from their inception to 30 June 2024. The search terms included “acute cholecystitis” and “Dachaihu decoction.” There were no language restrictions. The complete search strategy is given in Supplementary Material S2.

### 2.3 Research selection and data extraction

Two researchers (Xin-xin Liu and Ying-qi Ma) independently reviewed the literature, extracted data, and cross-checked the findings. Any disagreements were resolved by consulting a third reviewer (Jian-ping Liu). The initial screening involved reviewing titles and abstracts to filter out irrelevant studies, followed by a full-text review to decide on final inclusion. The extracted data covered the first author, year of publication, sample size, gender and age of participants, symptom onset, intervention types, treatment duration, and outcomes. In this study, species classification and validation of the phytomedicines in Dachaihu decoction were performed using the ConPhyMP tool to meet drug classification standards.

### 2.4 Assessment of the risk of bias

Two researchers (Xin-xin Liu and Ling-yao Kong) independently evaluated the risk of bias in the included studies using the Cochrane’s Risk of Bias 2.0 tools, and the results were cross-checked. Any disagreements were resolved through consultation with a third reviewer (Jian-ping Liu). The assessment criteria included random sequence generation, allocation concealment, blinding (of implementers, participants, and outcome evaluators), incomplete outcome data, selective reporting, and other potential bias sources. Risks were categorized as “low risk,” “high risk,” or “some concerns” based on this assessment.

### 2.5 Statistical analysis

A meta-analysis was conducted using Stata 17.0 software, with the results reported as 95% confidence intervals (95% CIs) and statistical significance defined as *p* < 0.05. (1) Heterogeneity across studies was assessed using the I^2^ test. A fixed-effect model was used when heterogeneity was low (*p* > 0.1; I^2^ < 50%), while a random-effects model was applied for high heterogeneity (*p* < 0.1; I^2^ > 50%). (2) For a dichotomous variable, the effect size was calculated using relative risk (RR), and for continuous variables, mean difference (MD) was used. When studies used different units of measurement, standardized mean difference (SMD) was applied.

### 2.6 Subgroup and sensitivity analyses

Subgroup analyses were carried out according to the type of intervention to evaluate the consistency and reliability of the results. Sensitivity analyses were used to assess whether the results and heterogeneity changed when a single study was excluded.

### 2.7 Publication bias

Publication bias was assessed if the funnel plot showed asymmetry or if the Egger test indicated bias (*p* < 0.05). In such cases, the “trim-and-fill” method was used to estimate the adjusted combined effect size.

### 2.8 Assessment of certainty of evidence

The certainty of evidence for an outcome measure was evaluated using the Grading of Recommendations Assessment, Development, and Evaluation (GRADE) assessment system. Two researchers (Xin-xin Liu and Ling-yao Kong) independently evaluated GRADE. Any disagreements were resolved by consulting a third reviewer (Jian-ping Liu). This evaluation considers five factors: risk of bias, consistency of results, indirectness of evidence, imprecision, and potential publication bias. Based on these criteria, the evidence was classified as “high,” “moderate,” “low,” or “very low” (Grade Working Group, 2017).

## 3 Results

### 3.1 Results of the literature search

**FIGURE 1:**
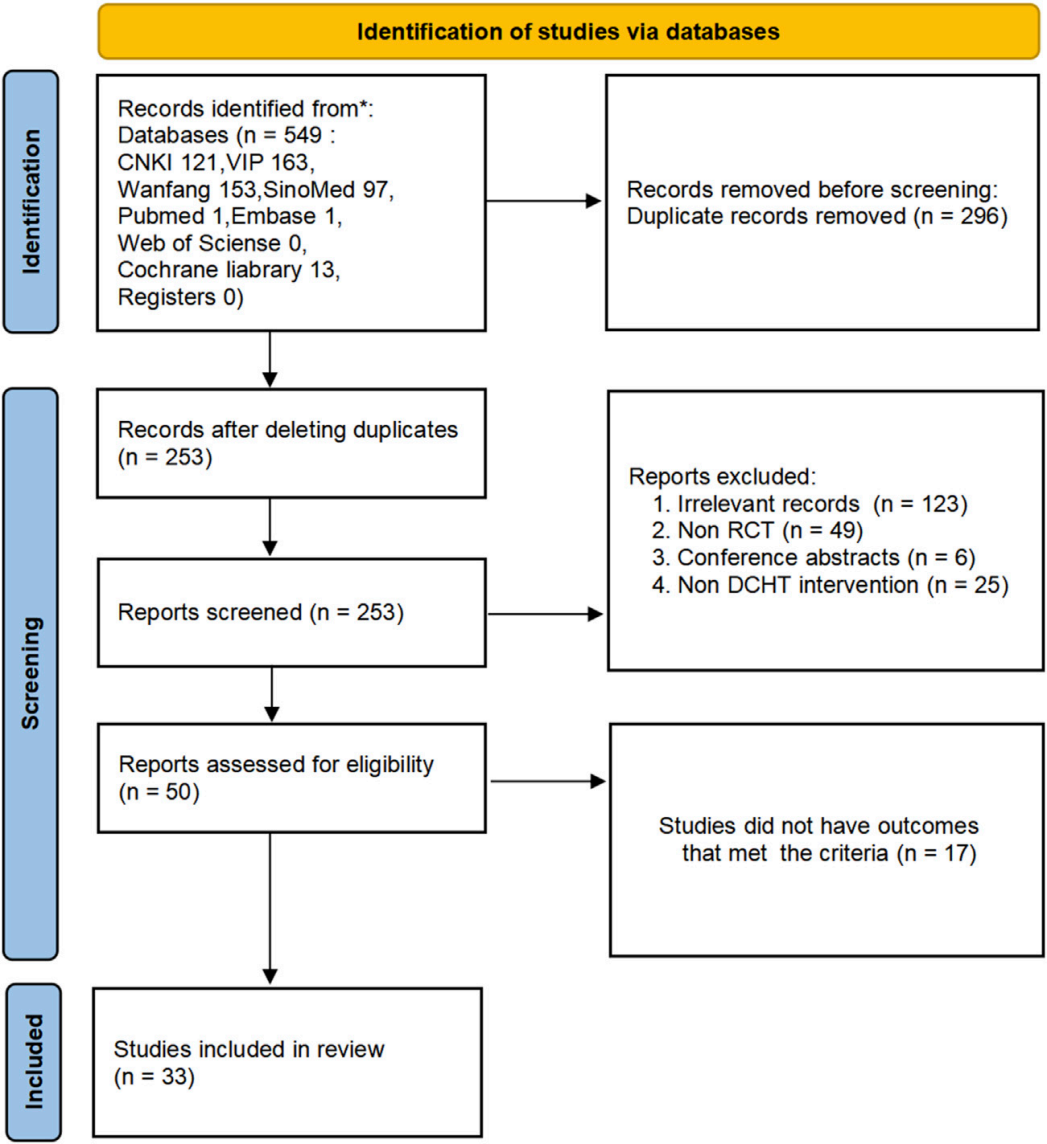
Flow diagram of the literature screening process.

A comprehensive computerized search retrieved 549 studies (121 studies in CNKI, 163 in VIP, 153 in Wanfang, 97 in SinoMed, 1 in PubMed, 1 in Embase, 0 in Web of Science, 13 in Cochrane Library, 0 in International Clinical Trials Registry Platform, 0 in ClinicalTrials.gov, and 0 in Chinese Clinical Trial Registry). A total of 296 duplicate records were removed, and full texts of the remaining 253 studies were reviewed. Studies that did not meet the inclusion criteria were excluded. Ultimately, 33 RCTs (Zhou, 2020; Liu et al., 2021; Cai, 2022; Chen et al., 2023; Deng, 2018; Deng et al., 2018; Gu, 2021; Guo, 2012; Guo, 2023; Hong and Gao, 2018; Li, 2016; Liang and Deng, 2013; Liang et al., 2013; Liao, 2014; Liu, 2021; Liu, 2015; Su, 2016; Sun, 2005; Wang et al., 2020; Wang and Li, 2014; Wang, 2010; Wei, 2016; Xia, 2020; Xiong and Yi, 2021; Xu, 2017; Yang, 2016; Yu, 2009; Zhang et al., 2003; Zhang, 2018; Zhou, 2019; Zhu, 2017; Zhuang, 2021; Zuo, 2021) were included. The flow diagram of the selection process is shown in Figure 1.

### 3.2 Study characteristics

The selected studies, published between 2003 and 2023, accounted for 81.81% (27/33) of the studies conducted in the past decade. The 33 RCTs included 2,851 Chinese patients diagnosed with AC, comprising 1,404 male and 1,447 female individuals. Sample sizes ranged from 40 to 206 cases. Nineteen studies did not specify the duration of the onset of illness, and the intervention was Dachaihu decoction or a combination of conventional treatment/LC, with the treatment duration ranging from 5 to 30 days. Outcomes included time to the disappearance of clinical symptoms, inflammation levels, reports of adverse events, clinical efficacy, length of hospital stay, and liver function. Study characteristics are given in Table 2.

**TABLE 2:** Study characteristics.

No.	Study ID	Sample size		Gender (male/female)		Age [mean (SD)]		Onset time (days)		Intervention		Duration (days)	Outcomes
		T	C	T	C	T	C	T	C	T	C		
1	Cai MK2022	103	103	48/55	47/56	51.24 ± 5.65	51.16 ± 5.72	8.14 ± 1.03	8.08 ± 1.10	DCHT + CT	CT	14	②, ③, ④, and ⑩
2	Chen JT2023	40	40	21/19	22/18	42.42 ± 4.74	42.37 ± 4.65	NR	NR	DCHT + LC	LC	5	①, ②, ③, ④, ⑤, ⑥, ⑦, and ⑩
3	Deng H2018	32	32	NR	NR	79.2 ± 6.8	80.4 ± 5.8	NR	NR	DCHT + CT	CT	14	② and ③
4	Deng YH2018	43	43	23/20	24/19	53.40 ± 13.50	53.60 ± 14.20	NR	NR	DCHT + CT	CT	28	①, ②, ③, and ⑧
5	Gu KK2021	30	30	17/13	16/14	62.71 ± 1.05	62.69 ± 1.06	0.29 ± 0.06	0.29 ± 0.05	DCHT + LC	LC	15	②, ③, ⑤, and ⑥
6	Guo AL2012	35	35	16/19	17/18	41.63 ± 10.21	40.23 ± 11.51	2.86 ± 1.35	3.0 ± 1.15	DCHT + CT	CT	7	⑤ and ⑩
7	Guo HL2023	40	40	17/23	16/24	56.34 ± 4.24	56.83 ± 4.57	NR	NR	DCHT + CT	CT	14	②, ③, ④, and ⑨
8	Hong JX2018	41	41	18/23	20/21	56.16 ± 10.23	54.02 ± 11.87	2.31 ± 0.86	2.46 ± 1.08	DCHT + CT	CT	7	⑦, ⑧, and ⑩
9	Li J2016	63	63	40/23	38/25	42.37 ± 5.63	43.68 ± 5.36	NR	NR	DCHT + CT	CT	10	①
10	Liang JB2013	50	50	32/18	30/20	47.00 ± 7.90	48.00 ± 8.30	1∼5	1∼6	DCHT	CT	10	①, ②, and ③
11	Liang JY2006	30	30	12/18	10/20	42.00 ± 11.20	43.52 ± 11.18	NR	NR	DCHT + CT	CT	7	② and ③
12	Liao XM2014	30	30	16/14	12/18	39.00 ± 3.70	38.00 ± 4.50	NR	NR	DCHT + CT	CT	30	②, ③, and ⑩
13	Liu H2021	35	35	19/16	18/17	52.34 ± 4.25	52.73 ± 4.59	3.02 ± 0.28	3.04 ± 0.26	DCHT + CT	CT	14	⑩
14	Liu WG2021	30	30	11/19	13/17	48.24 ± 8.13	48.71 ± 8.04	0.26 ± 0.05	0.28 ± 0.05	DCHT + LC	LC	14	①, ②, ③, ⑨, and ⑩
15	Liu YQ2015	52	52	22/30	21/31	53.63 ± 7.47	53.41 ± 7.62	3.01 ± 1.84	3.17 ± 1.96	DCHT + CT	CT	7	①, ②, ③, and ④
16	Su YB2016	41	41	20/21	17/24	39.65 ± 3.05	58.21 ± 4.35	NR	NR	DCHT + LC	LC	5	⑩
17	Sun JF2015	80	40	46/34	22/18	36∼65	38∼63	1∼5	1∼6	DCHT + CT	CT	10	①
18	Wang EC2020	40	40	26/14	22/18	NR	NR	2.00 ± 0.80	1.80 ± 0.60	DCHT + CT	CT	7	① and ⑥
19	Wang HR2014	59	59	25/34	27/32	39.58 ± 12.35	40.42 ± 11.92	NR	NR	DCHT + CT	CT	10	①, ②, ③, and ⑩
20	Wang SS2010	40	40	20/20	18/22	46.00 ± 10.80	40.60 ± 10.60	NR	NR	DCHT + CT	CT	7	② and ③
21	Wei YS2016	34	34	20/14	18/16	49.47 ± 8.13	50.34 ± 9.28	2.52 ± 0.96	2.47 ± 0.87	DCHT	CT	7	①, ②, ③, and ⑩
22	Xia CG2020	47	47	15/32	18/29	49.84 ± 16.92	48.05 ± 17.24	1.08 ± 0.47	1.00 ± 0.48	DCHT + LC	LC	10	②, ③, ④, ⑤, ⑥, and ⑦
23	Xiong Y2021	50	50	31/19	29/21	47.41 ± 9.81	48.28 ± 9.65	3.71 ± 1.37	3.45 ± 1.32	DCHT + CT	CT	5	②, ③, ⑧, and ⑩
24	Xu T2017	34	33	18/16	19/14	44.63 ± 7.81	45.37 ± 8.24	NR	NR	DCHT + LC	LC	5	③, ⑤, and ⑩
25	Yang DD2016	41	41	26/15	27/14	53.18 ± 5.32	52.34 ± 5.36	NR	NR	DCHT + CT	CT	14	②, ③, ④, and ⑩
26	Yu XJ2009	36	36	39/33		NR	NR	NR	NR	DCHT + CT	CT	20	①, ②, ③, and ④
27	Zhang JY2003	30	20	11/19	8/12	21∼65	19∼67	NR	NR	DCHT + CT	CT	5	⑥
28	Zhang N2018	45	45	21/24	22/23	71.32 ± 11.20	74.70 ± 13.90	NR	NR	DCHT + CT	CT	14	①, ⑥, and ⑨
29	Zhou H2019	20	20	10/10	11/9	54.17 ± 13.02	53.45 ± 13.19	NR	NR	DCHT + CT	CT	14	① and ⑥
30	Zhou MJ2020	38	38	23/15	22/16	41.60 ± 5.00	39.70 ± 5.10	NR	NR	DCHT + CT	CT	30	①, ②, ③, and ⑧
31	Zhu WB2017	50	50	27/23	26/24	76.50 ± 1.50	75.50 ± 2.50	NR	NR	DCHT	CT	14	⑩
32	Zhuang LL2021	40	40	18/22	15/25	43.57 ± 10.89	41.50 ± 8.65	NR	NR	DCHT + CT	CT	14	⑧ and ⑩
33	Zuo YZ2021	72	72	35/37	36/36	60.83 ± 60.83	59.83 ± 17.17	0.26 ± 0.12	0.28 ± 0.09	DCHT + CT	CT	7	⑥

Note: T, treatment group; C, control group; NR, not reported; CT, conventional treatment; DCHT, Dachaihu tang; LC, laparoscopic cholecystectomy; ① clinical efficacy; ② time for resolution of abdominal pain; ③ time for resolution of fever; ④ time for white blood cell counts to normalize; ⑤ length of hospital stay; ⑥ levels of alanine aminotransferase (ALT); ⑦ levels of aspartate aminotransferase (AST); ⑧ neutrophil percentage (NEUT%); ⑨ levels of tumor necrosis factor-α (TNF-α); ⑩ incidence of adverse events.^2^

### 3.3 Risk of bias assessments

**FIGURE 2:**
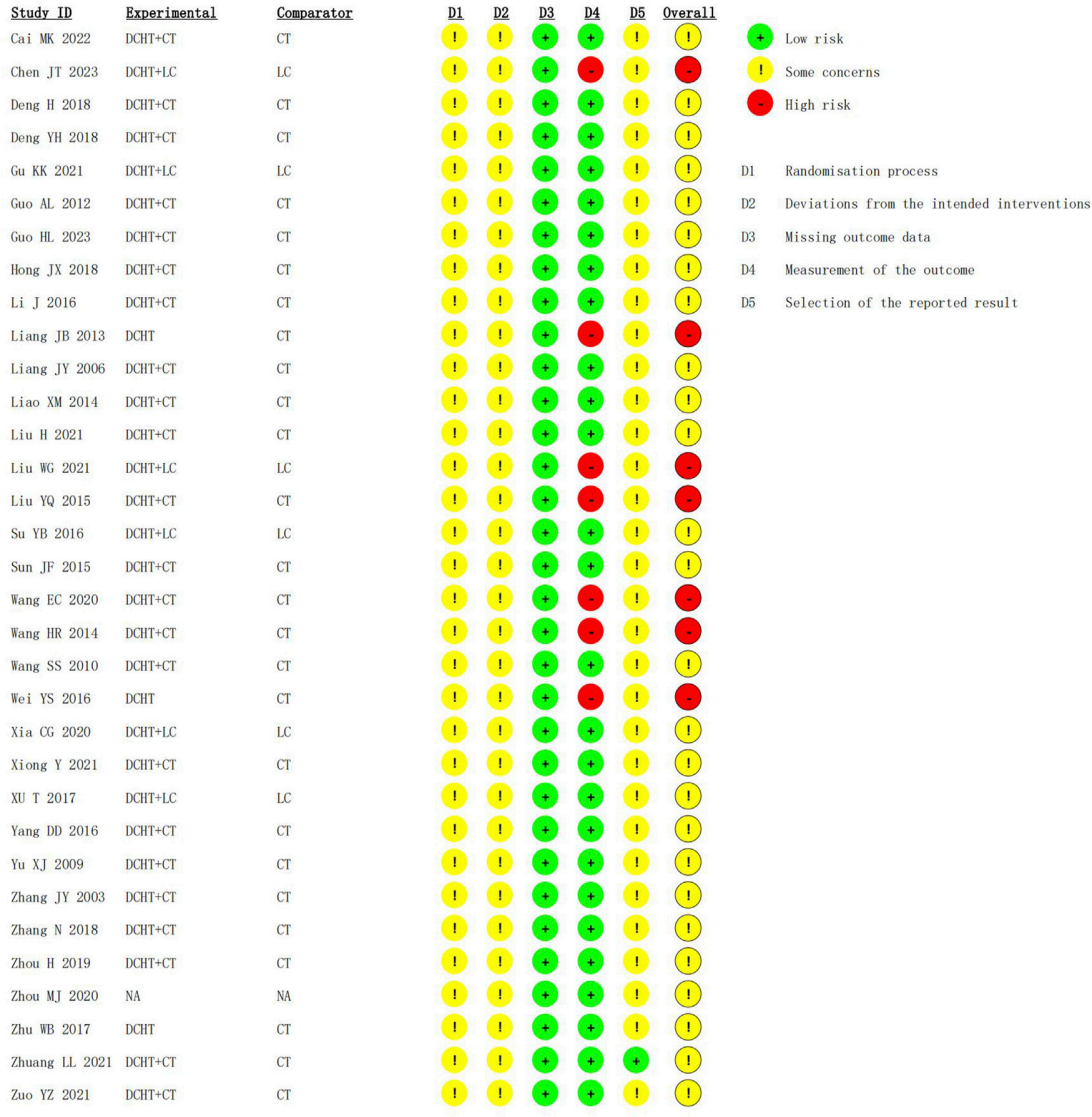
Risk of bias plot of included studies.

**FIGURE 3:**
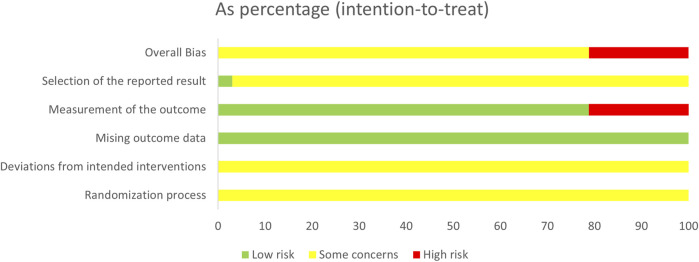
Summary of risk of bias in the included studies.

Among the 33 RCTs, 16 (Liu et al., 2021; Deng et al., 2018; Gu, 2021; Hong and Gao, 2018; Liao, 2014; Liu, 2021; Liu, 2015; Wang et al., 2020; Wang and Li, 2014; Xia, 2020; Xiong and Yi, 2021; Xu, 2017; Yang, 2016; Zhang, 2018; Zhu, 2017; Zhuang, 2021) used random number tables, 1 (Chen et al., 2023) utilized lotteries, and 1 (Zhou, 2020) adopted two-color ballots, while the remaining 15 merely mentioned “random.” All studies were comparable at baseline; however, none provided details on the implementation of allocation concealment, making it unclear whether it was properly executed. As such, this aspect was rated as having “some concerns” regarding randomization. Furthermore, none of the studies reported blinding of participants or outcome assessors, leading to a rating of “some concerns” for potential deviation from the intended intervention. Despite these limitations, all included studies presented complete data, thus receiving a “low-risk” rating for data completeness. In terms of outcome assessment, 26 studies were rated as “low risk,” whereas 7 (Liu et al., 2021; Li, 2016; Liang and Deng, 2013; Liu, 2015; Wang et al., 2020; Wang and Li, 2014; Wei, 2016) were considered “high risk” due to inappropriate measurement methods. Regarding selective reporting, only one study was rated as “low risk,” while the others lacked registration program documentation, resulting in a “some concerns” rating due to potential selective reporting bias (Figures 2, 3).

### 3.4 Meta-analysis of the results

#### 3.4.1 Clinical efficacy

**FIGURE 4:**
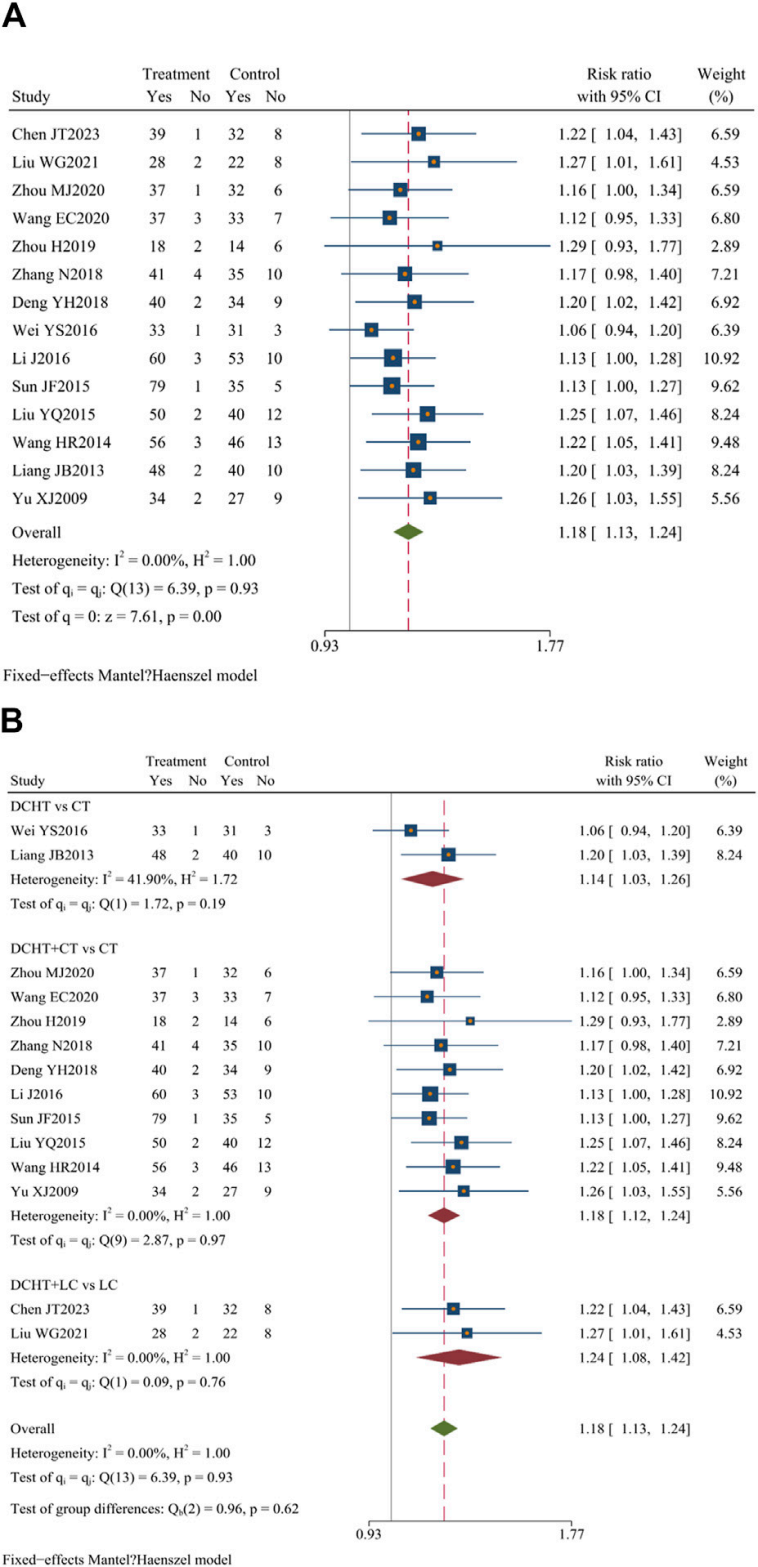
**(A)** Clinical efficacy, **(B)** Subgroup analysis of clinical efficacy. DCHT, Dachaihu tang; CT, Conventional treatment; LC, Laparoscopic cholecystectomy.

Among the 33 included studies, 14 (Zhou, 2020; Liu et al., 2021; Chen et al., 2023; Deng et al., 2018; Li, 2016; Liang and Deng, 2013; Liu, 2015; Sun, 2005; Wang et al., 2020; Wang and Li, 2014; Wei, 2016; Yu, 2009; Zhang, 2018; Zhou, 2019) (n = 1,219) assessed clinical efficacy, revealing a statistically significant difference between the groups (RR = 1.18; 95% CI = 1.13 to 1.24; I^2^ = 0.00%; Q (13) = 6.39; *p* = 0.00) (Figure 4A).

##### 3.4.1.1 Subgroup analysis of clinical efficacy

Subgroup analysis revealed that, compared with the CT group, the Dachaihu decoction group showed an improvement in clinical efficacy (RR = 1.14; 95% CI = 1.03 to 1.26; I^2^ = 41.90%). The combination of Dachaihu decoction and CT also showed notable improvements (RR = 1.18; 95% CI = 1.12 to 1.24; I^2^ = 0.00%). Similarly, combining Dachaihu decoction with LC significantly enhanced clinical efficacy (RR = 1.24; 95% CI = 1.08 to 1.42; I^2^ = 0.00%) Figure 4B.

#### 3.4.2 Time for resolution of abdominal pain

**FIGURE 5:**
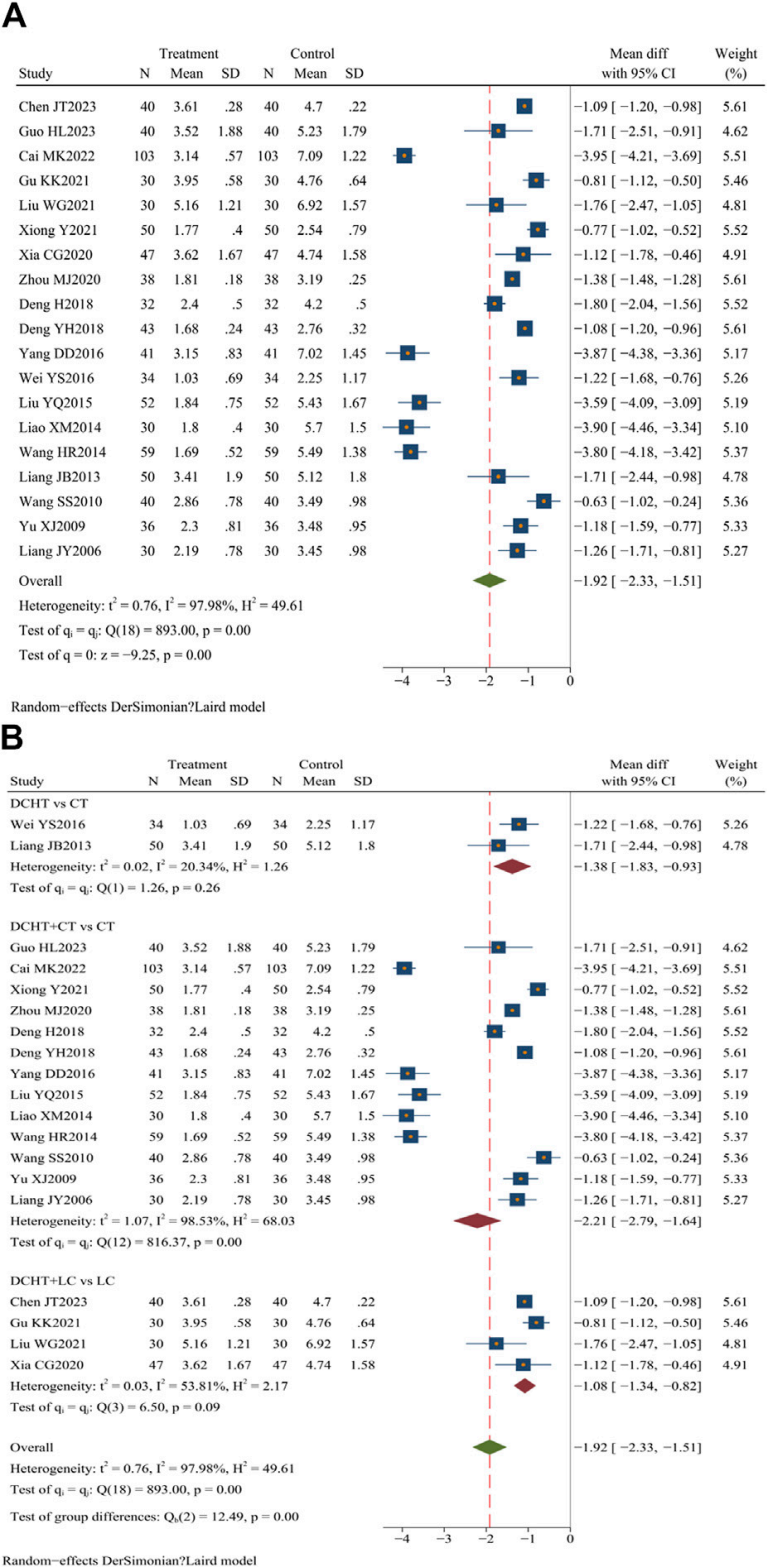
**(A)** Time for resolution of abdominal pain, **(B)** Subgroup analysis of time for resolution of abdominal pain. DCHT, Dachaihu tang; CT, Conventional treatment; LC, Laparoscopic cholecystectomy.

Nineteen studies (Zhou, 2020; Liu et al., 2021; Cai, 2022; Chen et al., 2023; Deng, 2018; Deng et al., 2018; Gu, 2021; Guo, 2023; Liang and Deng, 2013; Liang et al., 2013; Liao, 2014; Liu, 2015; Wang and Li, 2014; Wang, 2010; Wei, 2016; Xia, 2020; Xiong and Yi, 2021; Yang, 2016; Yu, 2009) (n = 1,648) evaluated the time required for abdominal pain to disappear. The results showed that the treatment group significantly shortened the time for abdominal pain to subside compared to the control group (MD = −1.92 days; 95% CI = –2.33 to −1.51; I^2^ = 97.98%; Q (18) = 893.00; *p* = 0.00) (Figure 5A).

##### 3.4.2.1 Subgroup analysis

Subgroup analysis demonstrated that the Dachaihu decoction group significantly reduced the time for abdominal pain relief compared to the CT group (MD = −1.38 days; 95% CI = –1.83 to −0.93; I^2^ = 20.34%). The Dachaihu decoction combined with CT also significantly reduced abdominal pain duration (MD = −2.21 days; 95% CI –2.79 to −1.64; I^2^ = 98.53%). When combined with LC, the decoction further reduced the time for abdominal pain to disappear (MD = −1.08 days; 95% CI = −1.34 to −0.82; I^2^ = 53.81%) (Figure 5B).

##### 3.4.2.2 Sensitivity analysis

In the Dachaihu decoction combined with CT compared to the CT group, we further analyzed the reason for heterogeneity. Two studies (Zhou, 2020; Liao, 2014) were excluded because the time of treatment was >28 days, one (Deng, 2018) was excluded as a result of patients being ≥80 years old, two (Cai, 2022; Liu, 2015) were excluded because the onset time was >3 days, and two studies (Liu et al., 2021; Xu, 2017) were excluded because they did not mention the type and dosage of anti-infective drugs. The heterogeneity was reduced when the above studies were excluded (I^2^ = 62%). There were still statistical differences (*p* < 0.05) (Supplementary Material S3).

#### 3.4.3 Time for resolution of fever

**FIGURE 6:**
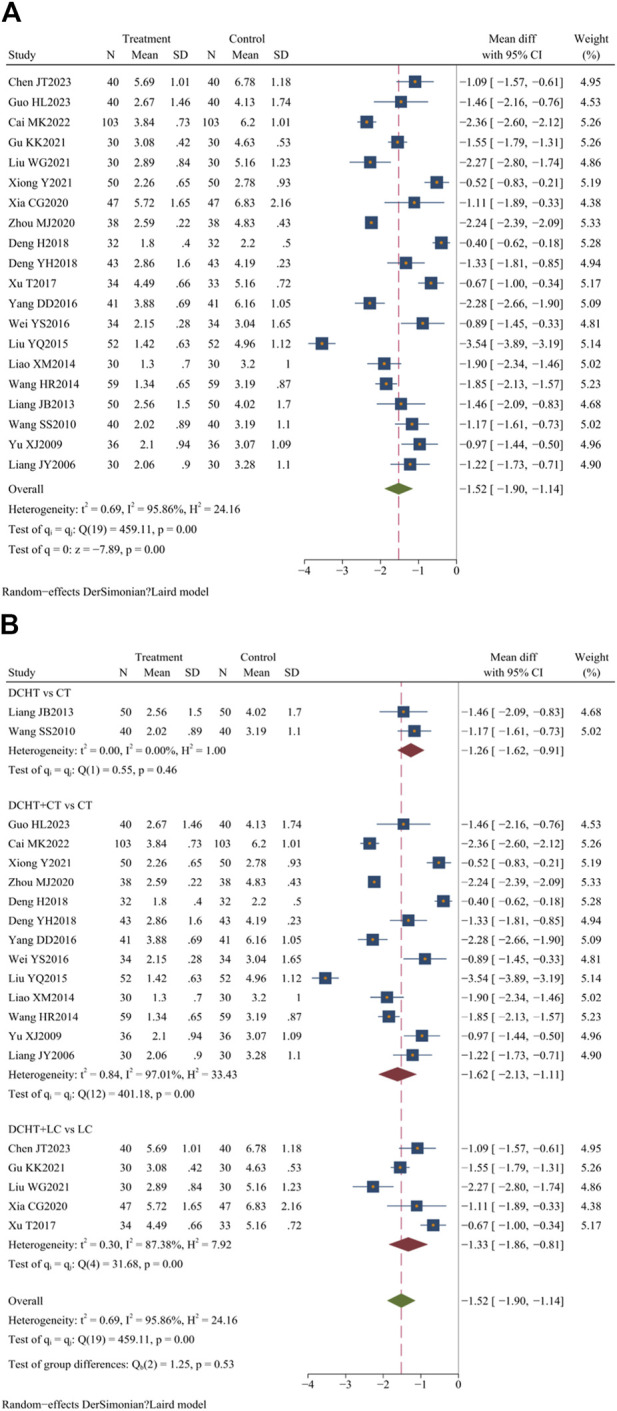
**(A)** Time for resolution of fever, **(B)** Subgroup analysis of time for resolution of fever. DCHT, Dachaihu tang; CT, Conventional treatment; LC, Laparoscopic cholecystectomy.

Twenty studies (Zhou, 2020; Liu et al., 2021; Cai, 2022; Chen et al., 2023; Deng, 2018; Deng et al., 2018; Gu, 2021; Guo, 2023; Liang and Deng, 2013; Liang et al., 2013; Liao, 2014; Liu, 2015; Wang and Li, 2014; Wang, 2010; Wei, 2016; Xia, 2020; Xiong and Yi, 2021; Xu, 2017; Yang, 2016; Yu, 2009) (n = 1717) assessed the time for fever resolution. Compared with the control group, the Dachaihu decoction group appeared to significantly shorten the duration of fever in patients (MD = −1.52 days; 95% CI = –1.90 to −1.14; I^2^ = 95.86%; Q (19) = 459.11; *p* = 0.00) (Figure 6A).

##### 3.4.3.1 Subgroup analysis

The subgroup analysis showed that compared with the CT group, the Dachaihu decoction group significantly reduced fever duration (MD = −1.26 days; 95% CI = –1.62 to −0.91; I^2^ = 0.00%). The combination of Dachaihu decoction and CT also significantly reduced fever duration (MD = −1.62 days; 95% CI = –2.13 to −1.11; I^2^ = 97.01%). When combined with LC, the duration of fever was significantly reduced (MD = −1.33 days; 95% CI = –1.86 to −0.81; I^2^ = 87.38%) (Figure 6B).

##### 3.4.3.2 Sensitivity analysis

Further analysis of heterogeneity was conducted. In the Dachaihu decoction combined with the CT group versus the CT group, two studies (Zhou, 2020; Guo, 2012) were excluded due to treatment durations exceeding 28 days, one study (Deng, 2018) was excluded due to the inclusion of patients aged ≥80 years, and three studies (Cai, 2022; Liu, 2015; Xiong and Yi, 2021) were excluded because the onset time exceeded 3 days, resulting in reduced heterogeneity (I^2^ = 79.00%). In the Dachaihu decoction combined with the LC group versus the LC group, two studies (Liu et al., 2021; Xu, 2017) were excluded because they did not mention the type and dosage of anti-infective drugs, significantly reducing heterogeneity (I^2^ = 40%). Statistical differences remained (*p* < 0.05) (Supplementary Material S3).

#### 3.4.4 Time for white blood cell counts to normalize

**FIGURE 7:**
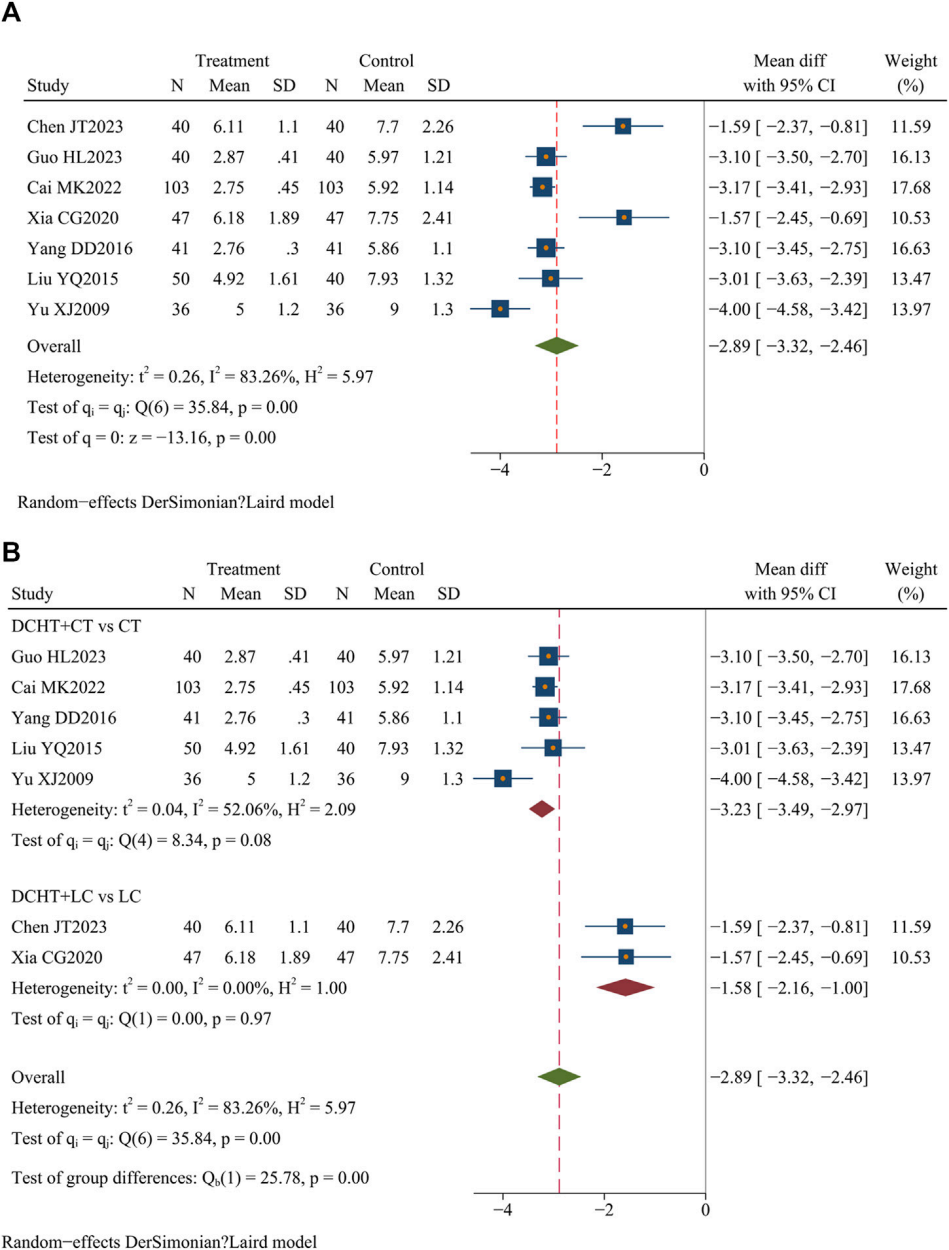
**(A)** Time for white blood cell counts to normalize, **(B)** Subgroup analysis of time for white blood cell counts to normalize. DCHT, Dachaihu tang; CT, Conventional treatment; LC, Laparoscopic cholecystectomy.

Of the 33 included studies, 7 (Cai, 2022; Chen et al., 2023; Guo, 2023; Liu, 2015; Xia, 2020; Yang, 2016; Yu, 2009) (n = 704) assessed the time required for white blood cell counts to normalize. The results showed that the treatment group significantly reduced the time compared to the control group (MD = −2.89 days; 95% CI = –3.32 to −2.46; I^2^ = 83.26%; Q (6) = 35.84; *p* = 0.00) (Figure 7A).

##### 3.4.4.1 Subgroup analysis

The subgroup analysis revealed that compared with the CT group, the Dachaihu decoction combined with CT significantly reduced the time required for white blood cell counts to normalize (MD = −3.23 days; 95% CI = –3.49 to −2.97; I^2^ = 52.06%). Compared with the LC group, the combination of Dachaihu decoction and LC also significantly shortened this time (MD = −1.58 days; 95% CI = –2.16 to −1.00; I^2^ = 0.00%) (Figure 7B).

##### 3.4.4.2 Sensitivity analysis

In the Dachaihu decoction combined with the CT group versus the CT group, one study (Yu, 2009) was excluded due to the treatment duration exceeding 14 days and publication before 2010, significantly reducing heterogeneity (I^2^ = 0.00%). The results remained statistically significant (*p* < 0.05) (Supplementary Material S3).

#### 3.4.5 Length of hospital stay

**FIGURE 8:**
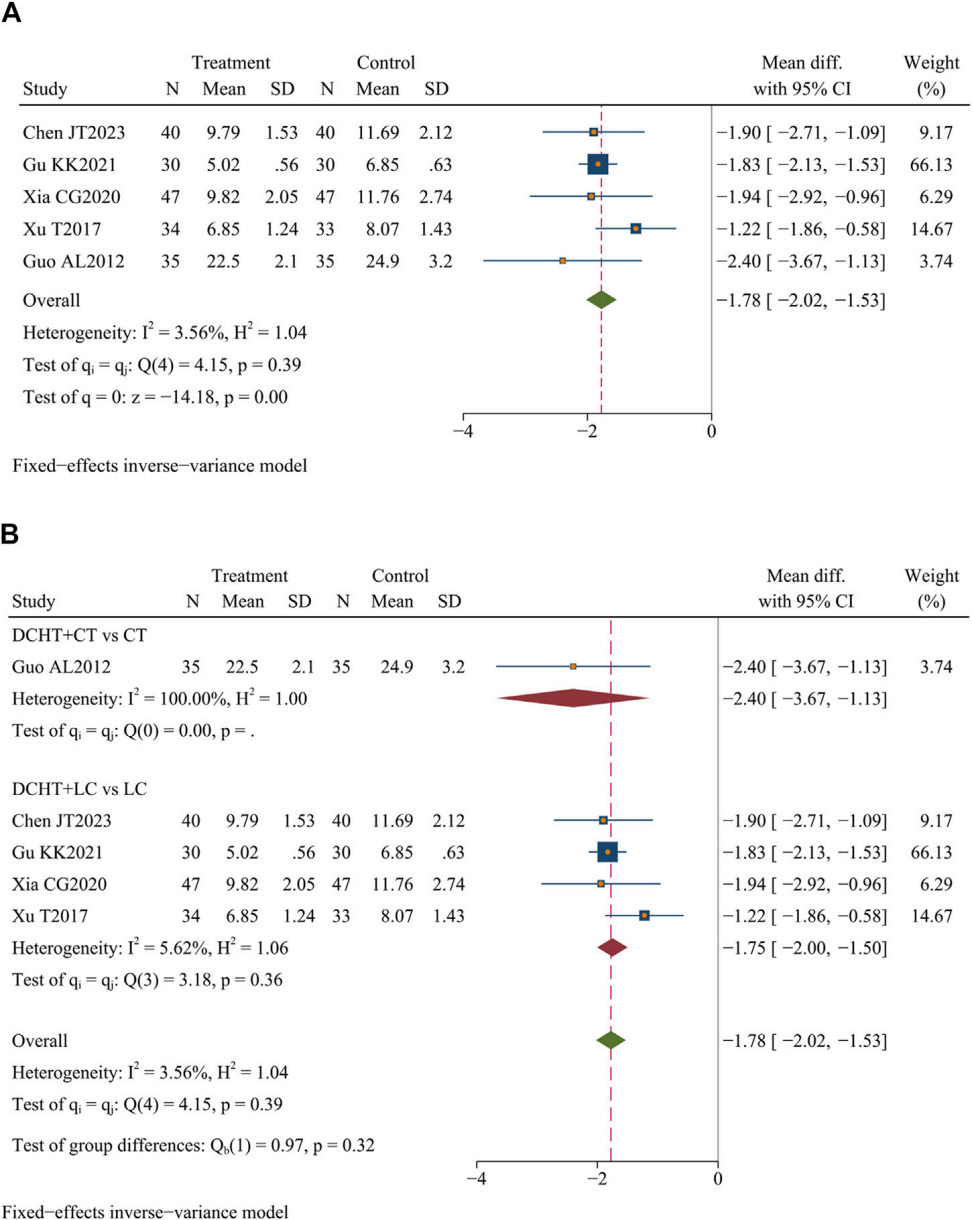
**(A)** Length of hospital stay, **(B)** Subgroup analysis of length of hospital stay. DCHT, Dachaihu tang; CT, Conventional treatment; LC, Laparoscopic cholecystectomy.

Five studies (Chen et al., 2023; Gu, 2021; Guo, 2012; Xia, 2020; Xu, 2017) (n = 371) assessed the length of stay. The results demonstrated that the Dachaihu decoction significantly shortened the length of hospital stay compared to the control group (MD = −1.78 days; 95% CI = –2.02 to −1.53; I^2^ = 3.56%; Q (4) = 4.15; *p* = 0.00) (Figure 8A).

##### 3.4.5.1 Subgroup analysis

Subgroup analysis revealed that compared with the LC, the combination of Dachaihu decoction and LC significantly shortened the length of hospital stay (MD = −1.75 days; 95% CI = –2.00 to −1.50; I^2^ = 5.62%) (Figure 8B).

#### 3.4.6 ALT levels

**FIGURE 9:**
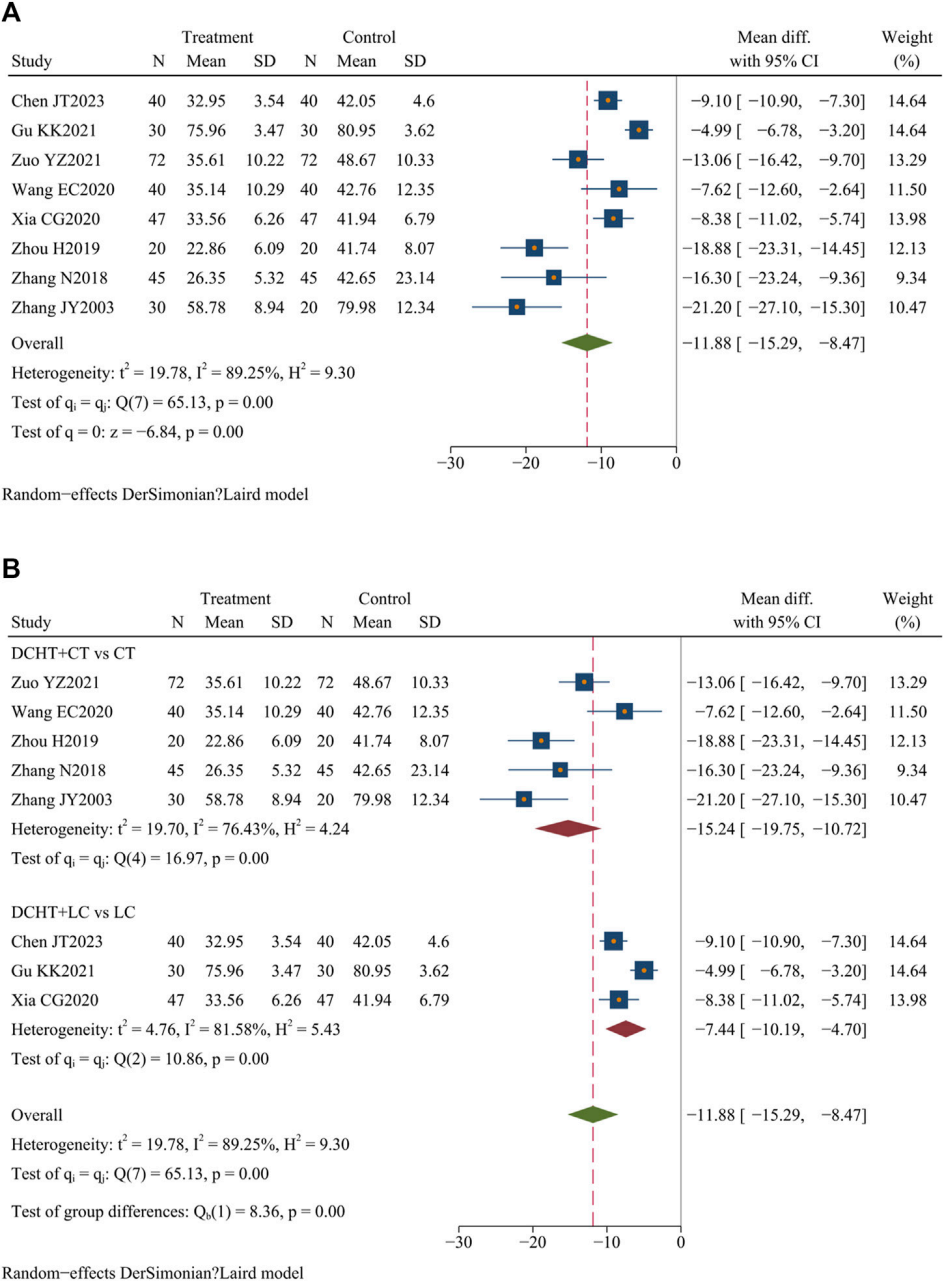
**(A)** ALT levels, **(B)** Subgroup analysis of ALT levels. DCHT, Dachaihu tang; CT, Conventional treatment; LC, Laparoscopic cholecystectomy.

Eight studies (Chen et al., 2023; Gu, 2021; Wang et al., 2020; Xia, 2020; Zhang et al., 2003; Zhang, 2018; Zhou, 2019; Zuo, 2021) (n = 638) assessed ALT levels. The results indicated that Dachaihu decoction significantly lowered ALT levels compared to the control group (MD = −11.88 U/L; 95% CI = –15.29 to −8.47; I^2^ = 89.25%; Q (7) = 65.13; *p* = 0.00) (Figure 9A).

##### 3.4.6.1 Subgroup analysis

The subgroup analysis indicated that the Dachaihu decoction combined with the CT group significantly reduced ALT levels compared with the CT group alone (MD = −15.24 U/L; 95% CI = –19.75 to −10.72; I^2^ = 76.43%). Dachaihu decoction combined with LC also significantly lowered ALT levels (MD = −7.44 U/L; 95% CI = −10.19 to −4.70; I^2^ = 81.58%) (Figure 9B).

##### 3.4.6.2 Sensitivity analysis

In the Dachaihu decoction combined with the CT group versus the CT group, heterogeneity was significantly reduced after excluding studies for specific reasons: one study (Wang et al., 2020) due to the mean age of included patients being <40 years, one (Zuo, 2021) for differing onset times, and one (Gu, 2021) because the included patients were elderly. Heterogeneity was significantly reduced (I^2^ = 0.00%), and the results remained statistically significant (*p* < 0.05) (Supplementary Material S3).

#### 3.4.7 AST levels

**FIGURE 10:**
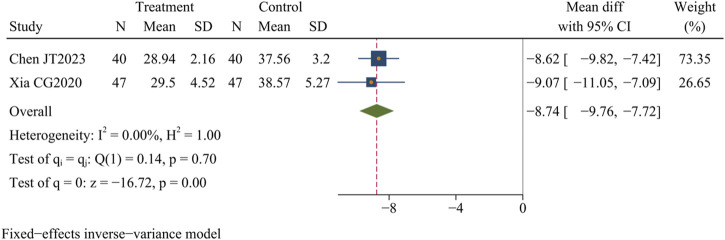
AST levels.

Two studies (Chen et al., 2023; Xia, 2020) (n = 174) evaluated the AST levels in patients with AC, and the findings showed that Dachaihu decoction significantly decreased AST levels compared to the control group (MD = −8.74 U/L; 95% CI = –9.76 to −7.72; I^2^ = 0.00%; Q (1) = 0.14; *p* = 0.00) (Figure 10).

#### 3.4.8 Neutrophil percentage

**FIGURE 11:**
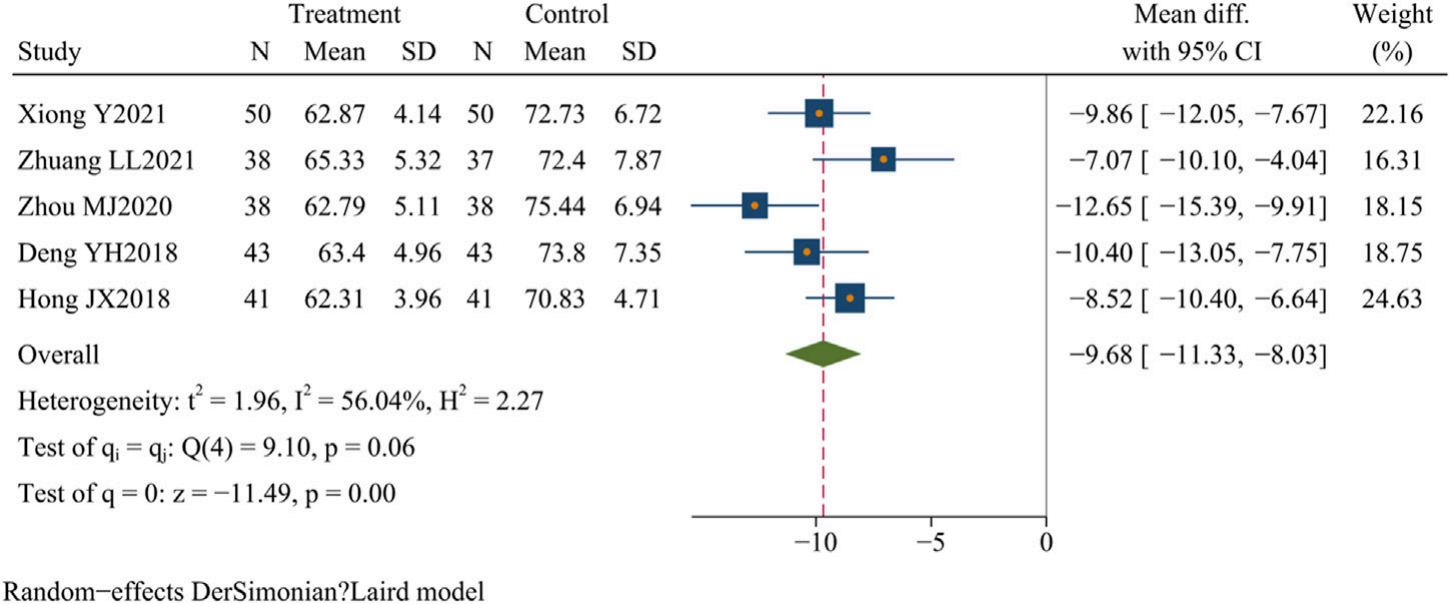
Neutrophil percentage.

Five studies (Deng et al., 2018; Hong and Gao, 2018; Xiong and Yi, 2021; Zhou, 2020; Zhuang, 2021) (n = 419) assessed neutrophil levels. The results demonstrated that Dachaihu decoction significantly reduced neutrophil levels compared with the control group (MD = −9.68; 95% CI = –11.33 to −8.03; I^2^ = 56.04%; Q (4) = 9.10; *p* = 0.00) (Figure 11).

#### 3.4.9 TNF-α levels

**FIGURE 12:**
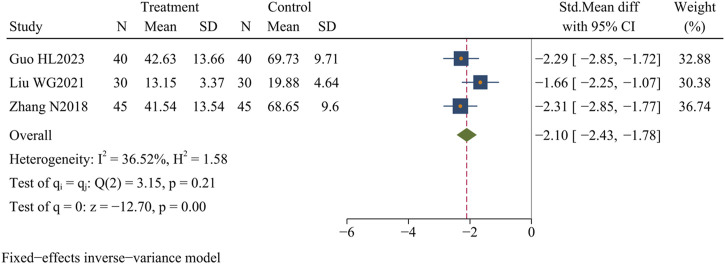
TNF-α levels.

Three studies (Liu et al., 2021; Guo, 2023; Zhang, 2018) (n = 230) assessed the TNF-α levels. The results showed that Dachaihu decoction significantly lowered TNF-α levels compared to the control group (SMD = −2.10 pg/L; 95% CI = –2.43 to −1.78; I^2^ = 36.52%; Q (2) = 3.15; *p* = 0.00) (Figure 12).

#### 3.4.10 Incidence of adverse events

**FIGURE 13:**
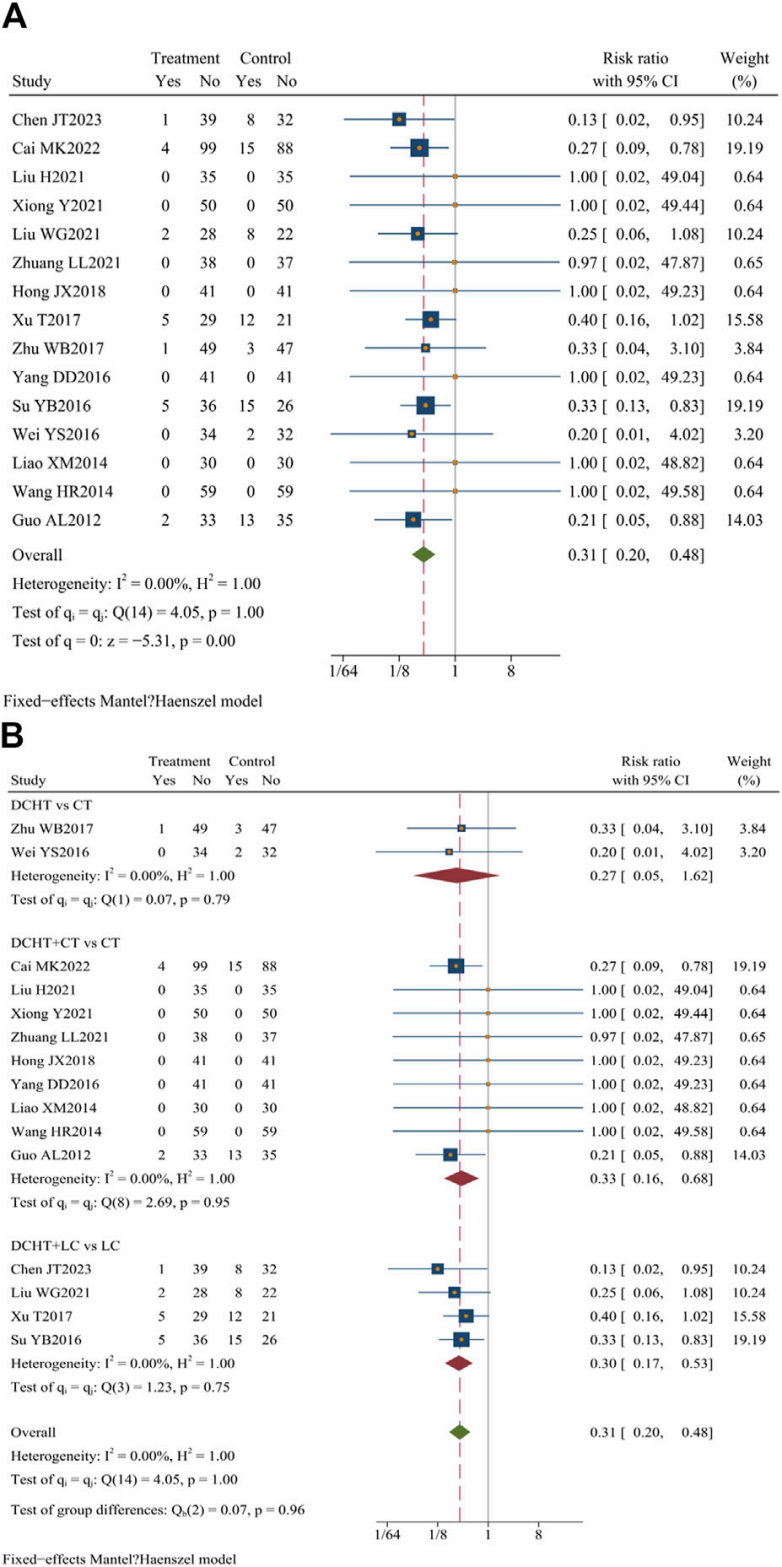
**(A)** Incidence of adverse events, **(B)** Subgroup analysis of incidence of adverse events. DCHT, Dachaihu tang; CT, Conventional treatment; LC, Laparoscopic cholecystectomy.

Fifteen studies (Liu et al., 2021; Cai, 2022; Chen et al., 2023; Guo, 2012; Hong and Gao, 2018; Liao, 2014; Liu, 2021; Su, 2016; Wang and Li, 2014; Wei, 2016; Xiong and Yi, 2021; Xu, 2017; Yang, 2016; Zhu, 2017; Zhuang, 2021) (n = 1,333) evaluated the incidence of adverse events. Cai (2022) and Guo (2012) indicated that the combination of Dachaihu decoction with CT was more effective in minimizing mild adverse reactions, such as diarrhea, nausea, insomnia, and palpitations, compared to using conventional treatment alone. Additionally, four studies (Liu et al., 2021; Chen et al., 2023; Su, 2016; Xu, 2017) observed that Dachaihu decoction combined with LC significantly reduced the occurrence of incision infection, biliary fistula, and bile duct injury. Furthermore, two studies (Wei, 2016; Zhu, 2017) found a lower incidence of mild adverse events, such as gastrointestinal discomfort, dizziness, and skin itching, in the Dachaihu decoction group than in routine treatment. In summary, Dachaihu decoction demonstrated better safety and tolerability in the treatment of acute cholecystitis, with potential benefits in reducing postoperative complications and alleviating gastrointestinal discomfort (RR = 0.31; 95% CI = 0.20 to 0.48; I^2^ = 0.00%; Q (14) = 4.05; *p* = 0.00) (Figure 13A; Table 3).

**TABLE 3:** Incidence of adverse events.

Comparison	Study ID	Adverse event category	Experimental group	Control group
DCHT + CT VS CT	Cai MK2022	Diarrhea	0 (0%)	2 (2%)
		Nausea	2 (2%)	6 (6%)
		Insomnia	1 (1%)	4 (4%)
		Palpitations	1 (1%)	3 (3%)
	Liu H2021	None	0 (0%)	0 (0%)
	Xiong Y2021	None	0 (0%)	0 (0%)
	Zhuang LL2021	None	0 (0%)	0 (0%)
	Hong JX2018	None	0 (0%)	0 (0%)
	Yang DD2016	None	0 (0%)	0 (0%)
	Liao XM2014	None	0 (0%)	0 (0%)
	Wang HR2014	None	0 (0%)	0 (0%)
	Guo AL2012	Gastrointestinal symptom	2 (2%)	13 (14%)
DCHT + LC VS LC	Chen JT2023	Incision/abdominal infection	1 (3%)	4 (10%)
		Biliary fistula	0 (0%)	2 (5%)
		Bile duct injury	0 (0%)	2 (5%)
	Liu WG2021	Liver abscess	1 (3%)	2 (6%)
		Incision infection	1 (3%)	4 (12%)
		Peritoneal abscess	0 (0%)	2 (6%)
	Xu T2017	Incision bleeding/infection and biliary fistula	5 (14%)	12 (36%)
	Su YB2016	Incision bleeding/infection and biliary fistula	5 (12%)	15 (36%)
DCHT VS CT	Zhu WB2017	Nausea	1 (2%)	3 (6%)
	Wei YS2016	Dizziness and gastrointestinal symptom	0 (0%)	1 (3%)
		Skin itching and redness	0 (0%)	1 (3%)

##### 3.4.10.1 Subgroup analysis

The subgroup analysis revealed that the Dachaihu decoction group significantly lowered the incidence of adverse events (RR = 0.27; 95% CI = 0.05 to 1.62; I^2^ = 0.00%). Compared with the CT group, the Dachaihu decoction combined with CT markedly decreased the incidence of adverse events (RR = 0.33; 95% CI = 0.16 to 0.68; I^2^ = 0.00%), including gastrointestinal symptoms, insomnia, and palpitations. Compared with the LC group, the Dachaihu decoction combined with LC significantly reduced the incidence of adverse events (surgical incision bleeding/infection, biliary fistula, bile duct injury, abdominal abscess, and fall) (RR = 0.30; 95% CI = 0.17 to 0.53; I^2^ = 0.00%). Seven studies (Hong and Gao, 2018; Liao, 2014; Liu, 2021; Wang and Li, 2014; Xiong and Yi, 2021; Yang, 2016; Zhuang, 2021) reported no adverse events (Figure 13B).

### 3.5 Assessment of publication bias

**FIGURE 14:**
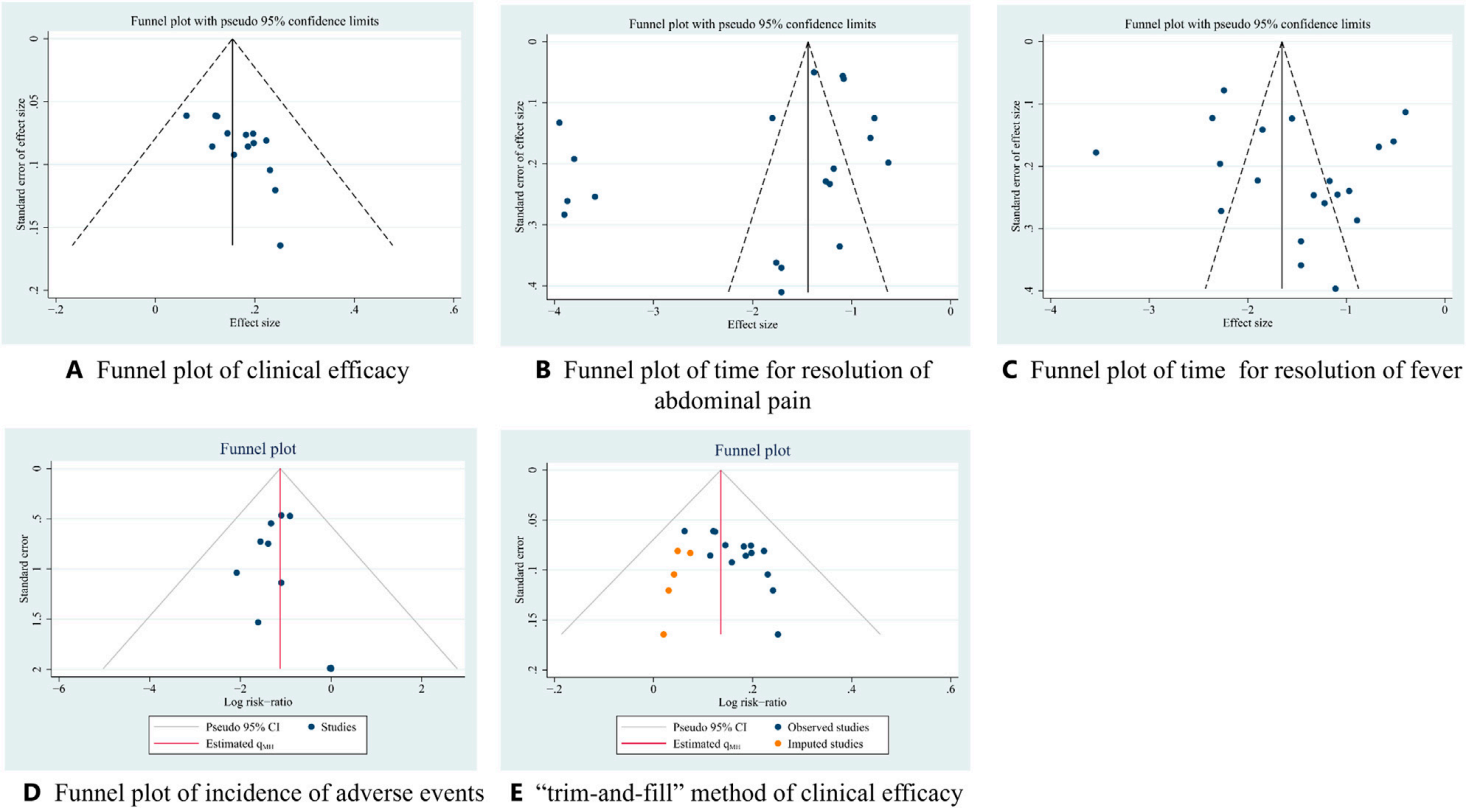
Funnel plot and “trim-and-fill” method.

As funnel plot analysis requires at least 10 original studies, this method was used to evaluate clinical efficacy, time required for the resolution of abdominal pain and fever, and the incidence of adverse events. Subsequent Egger’s test results indicated a significant difference in clinical efficacy (*p* < 0.05), suggesting publication bias. To address this, the “trim-and-fill” method was used to estimate combined effect sizes adjusted for publication bias. The adjusted pooled effect size remained significant (*p* < 0.01), suggesting that publication bias did not influence the assessment of clinical efficacy (Figures 14A–E).

### 3.6 Certainty of evidence

Certainty of evidence and the reasons for the upgrade and downgrade are presented in Table 4. The evidence for all outcomes was assessed as moderate to low certainty due to the risk of bias, imprecision, inconsistency, or indirectness.

**TABLE 4:** Certainty of evidence.

Quality assessment							No of patients		Effect		Quality	Importance
No. of studies	Design	Risk of bias	Inconsistency	Indirectness	Imprecision	Other considerations	Dachaihu decoction	Control	Relative (95% Cl)	Absolute		
Clinical efficacy												
14	Randomized trials	Serious^a^	No serious inconsistency	No serious indirectness	No serious imprecision	Reporting bias^b^	600/629 (95.4%)	474/590 (80.3%)	RR 1.18 (1.13–1.24)	145 more per 1,000 (from 104 more to 193 more)	⊕⊕○○ LOW	CRITICAL
								0%				
Time for resolution of abdominal pain (better indicated by lower values)												
19	Randomized trials	Serious^a^	Serious^c^	No serious indirectness	No serious imprecision	None	825	823	-	MD 1.92 lower (2.33–1.51 lower)	⊕⊕○○ LOW	IMPORTANT
Time for resolution of fever (better indicated by lower values)												
20	Randomized trials	Serious^a^	Serious^c^	No serious indirectness	No serious imprecision	None	859	858	-	MD 1.52 lower (1.90–1.14 lower)	⊕⊕○○ LOW	IMPORTANT
Time for white blood cell counts to normalize (better indicated by lower values)												
7	Randomized trials	Serious^a^	Serious^c^	No serious indirectness	No serious imprecision	None	357	347	-	MD 2.89 lower (3.32–2.46 lower)	⊕⊕○○ LOW	IMPORTANT
Length of hospital stay (better indicated by lower values)												
5	Randomized trials	Serious^a^	No serious inconsistency	No serious indirectness	Serious^d^	None	186	185	-	MD 1.78 lower (2.02–1.53 lower)	⊕⊕○○ LOW	CRITICAL
ALT levels (better indicated by lower values)												
8	Randomized trials	Serious^a^	Serious^c^	No serious indirectness	No serious imprecision	None	324	314	-	MD 11.88 lower (15.29–8.47 lower)	⊕⊕○○ LOW	IMPORTANT
AST levels (better indicated by lower values)												
2	Randomized trials	Serious^a^	No serious inconsistency	No serious indirectness	Serious^d^	None	87	87	-	MD 8.74 lower (9.76–7.72 lower)	⊕⊕○○ LOW	IMPORTANT
NEUT% (better indicated by lower values)												
5	Randomized trials	Serious^a^	Serious^c^	No serious indirectness	No serious imprecision	None	210	209	-	MD 9.68 lower (11.33–8.03 lower)	⊕⊕○○ LOW	IMPORTANT
TNF-α levels (better indicated by lower values)												
3	Randomized trials	Serious^a^	No serious inconsistency	No serious indirectness	Serious^d^	None	115	115	-	SMD 2.10 lower (2.43–1.78 lower)	⊕⊕○○ LOW	IMPORTANT
Incidence of adverse events												
15	Randomized trials	Serious^a^	No serious inconsistency	No serious indirectness	No serious imprecision	None	20/661 (3%)	76/672 (11.3%)	RR 0.31 (0.20–0.48)	78 fewer per 1,000 (from 59 fewer to 90 fewer)	⊕⊕⊕○ MODERATE	CRITICAL
								0%		-		

Note:

^a^ Have not implemented allocation concealment and blinding methods.

^b^ There is publication bias in this outcome.

^c^ High heterogeneity and little overlapping confidence intervals.

^d^ Sample size < 400.

## 4 Discussion

### 4.1 Main findings

A total of 33 eligible RCTs involving 2,851 participants evaluated the use of Dachaihu decoction alone or in combination with conventional treatment/LC for treating AC. Dachaihu decoction demonstrated potential benefits in improving clinical symptoms, shortening hospital stays, protecting liver function, reducing inflammatory responses, and decreasing adverse events (adverse drug reactions and postoperative complications). Subgroup analyses revealed that Dachaihu decoction alone may alleviate abdominal pain and fever, with no significant adverse events reported. When combined with CT or LC, it may further improve liver function, reduce inflammation, and enhance the symptoms of fever and abdominal pain in AC. Additionally, it may shorten the length of hospital stay, increase clinical efficacy, and decrease adverse event rates, indicating potential for clinical use. The sensitivity analysis showed that excluding different studies did not significantly alter the primary outcome’s direction or significance, suggesting consistent findings. Despite variations in study quality, the results remained applicable and interpretable. Due to an insufficient description of the randomization process and blinding design, these studies demonstrated a high risk of bias. No publication bias was observed, except in clinical efficacy. The evidence was assessed as having moderate-to-low certainty.

Classical TCM formulas (Dachaihu decoction) have distinct components and therapeutic properties. According to evidence-based TCM principles, clinicians can slightly adjust the grammage and composition of botanical drugs of Dachaihu decoction to meet individual patient needs. Multiple studies have shown that modifying Dachaihu decoction can improve symptom management through synergistic and complementary effects (Cheang et al., 2024; Dai et al., 2016; Li et al., 2014). The inclusion of multiple RCTs showed significant improvements in efficacy by customizing Dachaihu decoction’s grammage of certain botanical drugs to target the primary symptoms of diverse patients, underscoring its potential as an extension of the therapeutic system.

In the United States, approximately 20 million people experience AC annually, leading to healthcare costs exceeding $6.3 billion and imposing a substantial economic burden (Anderloni and Fugazza, 2022). A study reported that perioperative AC requires prolonged antibacterial treatment, which significantly increases hospitalization medical expenses and contributes to antibiotic resistance (Murata et al., 2013). The association of surgical infection is put forward for the quiet line in low-risk patients with laparoscopic cholecystectomy, not recommended for the routine use of perioperative antibiotics (Colling et al., 2022). Another study found that the complication rate associated with antibiotic therapy in elderly patients with AC was approximately 33% (Kivivuori et al., 2023). Our study found that the treatment of this disease with Dachaihu decoction might shorten the average length of hospital stay by nearly 2 days. This potential shortened the duration of symptoms but also reduced the nation’s health expenditure and the patients’ individual economic burden.

AC, a common inflammatory condition of the gallbladder, is characterized by inflammation and dyskinesia. Research has demonstrated that neutrophils contribute to both inflammation and gallbladder dysmotility in AC, potentially leading to gallbladder injury through the inhibition of SCF and c-kit expression (Zhou, 2020; Huang et al., 2016; Lin et al., 2019). TNF-α, a polymorphic hormone, is essential for regulating the body’s inflammatory and immune responses while promoting monocyte activity. It is involved in various local and systemic inflammatory reactions (Kalliolias and Ivashkiv, 2016). During AC episodes, a significant release of TNF-α occurs, resulting in the local inflammation of the gallbladder, fever, increased exudation, and right upper quadrant abdominal pain (Chen and Chen, 2023; Psaltis et al., 2024). This study suggested a potential reduction in the duration of abdominal pain and fever, neutrophil percentages, TNF-α levels, and white blood cell recovery times, following Dachaihu decoction treatment. These effects may be attributed to various active ingredients such as saikosaponin a, paeoniflorin, and aloe emodin, which inhibit IL-6, TNF-α, and CCK, thus modulating inflammation, enhancing immunity, repairing gallbladder damage, and promoting bile excretion and stone expulsion (Lin et al., 2019).

Dachaihu decoction reduces liver injury and enhances liver function through multiple mechanisms such as scavenging oxygen free radicals, reducing lipid peroxidation, promoting liver cell regeneration, and enhancing liver blood flow (Yang et al., 2019). AST, ALT, and serum total bilirubin (TBIL) are recognized as sensitive markers of hepatocyte injury. Extensive clinical and experimental research has demonstrated that Dachaihu decoction may significantly reduce serum AST and ALT levels, potentially aiding in liver function recovery and minimizing liver cell damage (Law et al., 2014; Han et al., 2016; Mou et al., 2024), which was consistent with our findings. This effect is likely mediated by the activation of the PI3K/AKT/STAT3 and PPARα signaling pathways, which regulate the expression of E-cadherin, N-cadherin, p53, Bax, Bcl-2, PI3K, p-AKT, AKT, STAT3, CYP7a1, and Cyp8b1, thereby reducing liver cell damage (Xu et al., 2022; Duan et al., 2024).

Antibiotics and non-steroidal anti-inflammatory drugs (NSAIDs) are frequently used to treat infections and relieve biliary colic. However, their use may increase the risk of severe and potentially life-threatening side effects, including gastrointestinal bleeding, kidney damage, and cardiovascular complications (Fraquelli et al., 2016). The risk of adverse events in perioperative patients ranges from 16% to 25% (Ishii et al., 2023), and significant controversy persists regarding the risk associated with different surgical protocols (Do et al., 2023; Gurusamy and Samraj, 2006; Cucchetti et al., 2022). Dachaihu decoction has been well-documented in the TCM literature, indicating a long-standing history of safe use. Several studies have shown that the eight botanicals in its formula usually have a better safety profile under conventional dosage (grams) (Yang et al., 2017; Song et al., 2020; Ali et al., 2008; Bai et al., 2022). Clinical reports showed that adverse reactions were typically mild gastrointestinal symptoms, which usually resolved on their own without the need for special intervention. Furthermore, Dachaihu decoction exhibits hepatoprotective effects in alpha-naphthyl isothiocyanate (ANIT)-induced liver injury models (Chen et al., 2016). Recent studies further demonstrate that Dachaihu decoction has protective effects on liver and kidney function, indicating a relatively safe profile (Huang et al., 2023; Qiao et al., 2022). However, while most clinical trials on drug interactions have highlighted its effects in reducing toxicity and enhancing efficacy, experimental research remains relatively limited, underscoring the need for careful monitoring during clinical use. Although some studies suggested that Dachaihu decoction did not increase adverse reactions and might have reduced complications when used preoperatively, a comprehensive assessment is still necessary. Current toxicity data are mostly derived from small-scale studies, lacking large-scale randomized controlled trials. More research is needed to further evaluate its safety and inform clinical applications.

## 5 Strengths and limitations

This study performed a systematic search across eight databases and three clinical trial registries to ensure broad literature coverage. A language-unrestricted search strategy was used to minimize selection bias. A total of 33 RCTs involving 2,851 patients were included, providing sufficient evidence to evaluate the efficacy and safety of Dachaihu decoction in treating acute cholecystitis. In terms of outcome measures, this study assessed a variety of indicators, such as clinical efficacy, time to resolution of abdominal pain and fever, time to white blood cell normalization, length of hospital stay, liver function, and inflammatory markers. These diverse measures allowed for a comprehensive evaluation of Dachaihu decoction’s effects, showcasing its potential multi-target and multi-mechanism benefits in managing acute cholecystitis. In addition, Dachaihu decoction is widely used in clinical practice and generally has a higher safety profile. Subgroup and sensitivity analyses were also conducted to identify the sources of heterogeneity and verify the stability of the results, thereby enhancing the findings’ reliability. The GRADE assessment system was utilized to evaluate the quality of evidence, offering a structured framework for interpreting results and guiding clinical decision-making and future research.

Nonetheless, our study has several limitations. First, the included studies were all conducted in China and involved only Chinese patients, which may introduce regional publication bias; therefore, additional international clinical trials are recommended. Second, the risk-of-bias assessment showed that most studies reported random sequence generation, but most lacked proper blinding or did not report it. It is essential to recognize that this methodological issue presents a frequent obstacle in many investigations of botanical drug tonics. Given that these tonics are mainly used in practical clinical environments, achieving full implementation of double-blind methods and allocation concealment is often difficult. Furthermore, the complex nature of botanical drug formulations, combined with the distinctive smells or colors of certain botanical drugs, adds to the challenge of blinding. To determine the potential influence of bias on study outcomes, a sensitivity analysis was performed, showing that the results maintained a reasonable degree of credibility. To improve the validity and consistency of future research on botanical drugs of tonic effectiveness, researchers are encouraged to apply stricter randomization and blinding techniques. Additionally, the control groups in these studies were limited to traditional modern medicine, with no placebo controls, and most studies did not include a follow-up period. Moreover, the GRADE assessment system found that most studies were of low quality, indicating a need for better quality and improved methods in current randomized controlled trials. The small sample size in this study may limit the generalizability and reliability of the results. Therefore, larger-scale studies are needed to confirm these findings. We also suggest that future researchers focus on implementing blinding and registering clinical trials to enhance study quality and credibility.

## 6 Conclusion

Dachaihu decoction may provide benefits in treating acute cholecystitis, such as reducing the length of hospital stay and improving symptoms, with a lower incidence of adverse events. However, due to the varying quality of the included studies and potential bias and heterogeneity, the accuracy of these findings may be limited. Caution is needed when interpreting the results. Future high-quality, large-scale, multi-center studies are necessary to further confirm the efficacy and safety of Dachaihu decoction for acute cholecystitis.

## Data Availability

The original contributions presented in the study are included in the article/Supplementary Material; further inquiries can be directed to the corresponding author.
