# Supplementary material for: Unveiling the therapeutic role of Dachaihu decoction in acute cholecystitis: a comprehensive systematic review and meta-analysis of its efficacy and safety
Source: Front Pharmacol. 2024 Nov 27;15:1497072. doi: 10.3389/fphar.2024.1497072 (PMC11631619; doi:10.3389/fphar.2024.1497072)
Supplement: Supplementary file 3 [file DataSheet3.docx]

Supplementary Materials

Article Title

Xin-xin Liu^1^, Ying-qi Ma^2^, Ling-yao Kong^1^, You-zhu Su^1^, Nicola Robinson^1,3^, Jian-ping Liu^1,4*^

1 Centre for Evidence-Based Chinese Medicine, Beijing University of Chinese Medicine, Beijing 100029, China

2 The First Clinical Medical College, Shandong University of Traditional Chinese Medicine, Jinan, Shandong, 252800, China

3 School of Health and Social Care, London South Bank University, London, SE1 0AA, UK

4 The National Research Center in Complementary and Alternative Medicine (NAFKAM), Department of Community Medicine, Faculty of Health Science, UiT The Arctic University of Norway, Tromsø, Norway

Correspondence author: Jian-ping Liu^*^Centre for Evidence Based Chinese Medicine, Beijing University of Chinese Medicine, Beijing 100029, China. E-mail address: Liujp@bucm.edu.cn

# Supplementary Material 1

Table 1 Information on the Dachaihu decoction in the included RCTs

| No. | Study ID | botanical drugs and dosage (g) of Dachaihu decoction | Preparation Method | Quality control standards for botanical drugs | Reported |
| --- | --- | --- | --- | --- | --- |
| 1 | Chen JT2023 | *Bupleurum chinense DC. [Apiaceae; Bupleuri radix]*(15g)、*Rheum officinale Baill. [Polygonaceae; Rhei radix et rhizoma]*(10g)、*Citrus aurantium L. [Rutaceae; Citri aurantii fructus immaturus]*(12g)、*Scutellaria baicalensis Georgi [Lamiaceae; Scutellariae radix]*(15g)、*Pinellia ternata (Thunb.) Makino [Araceae; Pinelliae rhizoma](Thunb.) Makino*.(6g)、*Paeonia lactiflora Pall. [Paeoniaceae; Paeoniae radix alba]*(10g)、*Zingiber officinale Roscoe [Zingiberaceae; Zingiberis rhizoma]*(15g)、*Ziziphus jujuba Mill. [Rhamnaceae; Ziziphi jujubae fructus]*(10g) | Prepared in accordance with the standards of the Chinese Pharmacopoeia and decocted in water. | Quality control standards for botanical drugs in compliance with the Chinese Pharmacopoeia | Yes |
| 2 | Cai MK2022 | *Bupleurum chinense DC. [Apiaceae; Bupleuri radix]*(10g)、*Rheum officinale Baill. [Polygonaceae; Rhei radix et rhizoma]*(10g)、*Citrus aurantium L. [Rutaceae; Citri aurantii fructus immaturus]*(10g)、*Scutellaria baicalensis Georgi [Lamiaceae; Scutellariae radix]*(10g)、*Paeonia lactiflora Pall. [Paeoniaceae; Paeoniae radix alba]*(10g)、*Zingiber officinale Roscoe [Zingiberaceae; Zingiberis rhizoma]*(10g)、 | Prepared in accordance with the standards of the Chinese Pharmacopoeia and decocted in water. | Quality control standards for botanical drugs in compliance with the Chinese Pharmacopoeia | Yes |
| 3 | Deng H2018 | *Bupleurum chinense DC. [Apiaceae; Bupleuri radix]*(9g)、*Rheum officinale Baill. [Polygonaceae; Rhei radix et rhizoma]*(3g)、*Citrus aurantium L. [Rutaceae; Citri aurantii fructus immaturus]*(9g)、*Scutellaria baicalensis Georgi [Lamiaceae; Scutellariae radix]*(9g)、*Pinellia ternata (Thunb.) Makino [Araceae; Pinelliae rhizoma](Thunb.) Makino*.(6g)、*Paeonia lactiflora Pall. [Paeoniaceae; Paeoniae radix alba]*(9g)、*Zingiber officinale Roscoe [Zingiberaceae; Zingiberis rhizoma]*(6g)、*Ziziphus jujuba Mill. [Rhamnaceae; Ziziphi jujubae fructus]*(9g) | Prepared in accordance with the standards of the Chinese Pharmacopoeia and decocted in water. | Quality control standards for botanical drugs in compliance with the Chinese Pharmacopoeia | Yes |
| 4 | Deng YH2018 | *Bupleurum chinense DC. [Apiaceae; Bupleuri radix]*(15g)、*Rheum officinale Baill. [Polygonaceae; Rhei radix et rhizoma]*(10g)、*Citrus aurantium L. [Rutaceae; Citri aurantii fructus immaturus]*(15g)、*Scutellaria baicalensis Georgi [Lamiaceae; Scutellariae radix]*(15g)、*Pinellia ternata (Thunb.) Makino [Araceae; Pinelliae rhizoma](Thunb.) Makino*.(15g)、*Paeonia lactiflora Pall. [Paeoniaceae; Paeoniae radix alba]*(10g)、*Zingiber officinale Roscoe [Zingiberaceae; Zingiberis rhizoma]*(15g)、*Ziziphus jujuba Mill. [Rhamnaceae; Ziziphi jujubae fructus]*(10g) | Prepared in accordance with the standards of the Chinese Pharmacopoeia and decocted in water. | Quality control standards for botanical drugs in compliance with the Chinese Pharmacopoeia | Yes |
| 5 | Gu KK2021 | *Bupleurum chinense DC. [Apiaceae; Bupleuri radix]*(10g)、*Rheum officinale Baill. [Polygonaceae; Rhei radix et rhizoma]*(3g)、*Citrus aurantium L. [Rutaceae; Citri aurantii fructus immaturus]*(15g)、*Scutellaria baicalensis Georgi [Lamiaceae; Scutellariae radix]*(10g) | Prepared in accordance with the standards of the Chinese Pharmacopoeia and decocted in water. | Quality control standards for botanical drugs in compliance with the Chinese Pharmacopoeia | Yes |
| 6 | Guo AL2012 | *Bupleurum chinense DC. [Apiaceae; Bupleuri radix]*(15g)、*Rheum officinale Baill. [Polygonaceae; Rhei radix et rhizoma]*(10g)、*Citrus aurantium L. [Rutaceae; Citri aurantii fructus immaturus]*(10g)、*Scutellaria baicalensis Georgi [Lamiaceae; Scutellariae radix]*(10g)、*Pinellia ternata (Thunb.) Makino [Araceae; Pinelliae rhizoma](Thunb.) Makino*.(10g)、*Paeonia lactiflora Pall. [Paeoniaceae; Paeoniae radix alba]*(15g)、*Zingiber officinale Roscoe [Zingiberaceae; Zingiberis rhizoma]*(10g) | Prepared in accordance with the standards of the Chinese Pharmacopoeia and decocted in water. | Quality control standards for botanical drugs in compliance with the Chinese Pharmacopoeia | Yes |
| 7 | Guo HL2023 | *Bupleurum chinense DC. [Apiaceae; Bupleuri radix]*(15g)、*Rheum officinale Baill. [Polygonaceae; Rhei radix et rhizoma]*(10g)、*Citrus aurantium L. [Rutaceae; Citri aurantii fructus immaturus]*(10g)、*Scutellaria baicalensis Georgi [Lamiaceae; Scutellariae radix]*(10g)、*Pinellia ternata (Thunb.) Makino [Araceae; Pinelliae rhizoma](Thunb.) Makino*.(10g)、*Paeonia lactiflora Pall. [Paeoniaceae; Paeoniae radix alba]*(10g) | Prepared in accordance with the standards of the Chinese Pharmacopoeia and decocted in water. | Quality control standards for botanical drugs in compliance with the Chinese Pharmacopoeia | Yes |
| 8 | Hong JX2018 | *Bupleurum chinense DC. [Apiaceae; Bupleuri radix]*(15g)、*Rheum officinale Baill. [Polygonaceae; Rhei radix et rhizoma]*(8g)、*Citrus aurantium L. [Rutaceae; Citri aurantii fructus immaturus]*(12g)、*Scutellaria baicalensis Georgi [Lamiaceae; Scutellariae radix]*(12g)、*Pinellia ternata (Thunb.) Makino [Araceae; Pinelliae rhizoma](Thunb.) Makino*.(10g)、*Paeonia lactiflora Pall. [Paeoniaceae; Paeoniae radix alba]*(15g)、*Ziziphus jujuba Mill. [Rhamnaceae; Ziziphi jujubae fructus]*(4 pieces) | Prepared in accordance with the standards of the Chinese Pharmacopoeia and decocted in water. | Quality control standards for botanical drugs in compliance with the Chinese Pharmacopoeia | Yes |
| 9 | Li J2016 | *Bupleurum chinense DC. [Apiaceae; Bupleuri radix]*(15g)、*Rheum officinale Baill. [Polygonaceae; Rhei radix et rhizoma]*(10g)、*Citrus aurantium L. [Rutaceae; Citri aurantii fructus immaturus]*(10g)、*Scutellaria baicalensis Georgi [Lamiaceae; Scutellariae radix]*(15g)、*Pinellia ternata (Thunb.) Makino [Araceae; Pinelliae rhizoma](Thunb.) Makino*.(10g)、*Paeonia lactiflora Pall. [Paeoniaceae; Paeoniae radix alba]*(15g)、*Zingiber officinale Roscoe [Zingiberaceae; Zingiberis rhizoma]*(3 slices)、*Ziziphus jujuba Mill. [Rhamnaceae; Ziziphi jujubae fructus]*(4 pieces) | Prepared in accordance with the standards of the Chinese Pharmacopoeia and decocted in water. | Quality control standards for botanical drugs in compliance with the Chinese Pharmacopoeia | Yes |
| 10 | Liang JB2013 | *Bupleurum chinense DC. [Apiaceae; Bupleuri radix]*(45g)、*Rheum officinale Baill. [Polygonaceae; Rhei radix et rhizoma]*(15g)、*Citrus aurantium L. [Rutaceae; Citri aurantii fructus immaturus]*(10g)、*Scutellaria baicalensis Georgi [Lamiaceae; Scutellariae radix]*(10g)、*Pinellia ternata (Thunb.) Makino [Araceae; Pinelliae rhizoma](Thunb.) Makino*.(10g)、*Paeonia lactiflora Pall. [Paeoniaceae; Paeoniae radix alba]*(30g) | Prepared in accordance with the standards of the Chinese Pharmacopoeia and decocted in water. | Quality control standards for botanical drugs in compliance with the Chinese Pharmacopoeia | Yes |
| 11 | Liang JY2006 | *Bupleurum chinense DC. [Apiaceae; Bupleuri radix]*(12g)、*Rheum officinale Baill. [Polygonaceae; Rhei radix et rhizoma]*(10g)、*Citrus aurantium L. [Rutaceae; Citri aurantii fructus immaturus]*(10g)、*Scutellaria baicalensis Georgi [Lamiaceae; Scutellariae radix]*(10g)、*Pinellia ternata (Thunb.) Makino [Araceae; Pinelliae rhizoma](Thunb.) Makino*.(10g)、*Paeonia lactiflora Pall. [Paeoniaceae; Paeoniae radix alba]*(15g) | Prepared in accordance with the standards of the Chinese Pharmacopoeia and decocted in water. | Quality control standards for botanical drugs in compliance with the Chinese Pharmacopoeia | Yes |
| 12 | Liao XM2014 | *Bupleurum chinense DC. [Apiaceae; Bupleuri radix]*(10g)、*Rheum officinale Baill. [Polygonaceae; Rhei radix et rhizoma]*(20g)、*Citrus aurantium L. [Rutaceae; Citri aurantii fructus immaturus]*(10g)、*Scutellaria baicalensis Georgi [Lamiaceae; Scutellariae radix]*(20g)、*Pinellia ternata (Thunb.) Makino [Araceae; Pinelliae rhizoma](Thunb.) Makino*.(15g)、*Paeonia lactiflora Pall. [Paeoniaceae; Paeoniae radix alba]*(20g) | Prepared in accordance with the standards of the Chinese Pharmacopoeia and decocted in water. | Quality control standards for botanical drugs in compliance with the Chinese Pharmacopoeia | Yes |
| 13 | Liu H2021 | *Bupleurum chinense DC. [Apiaceae; Bupleuri radix]*(15g)、*Citrus aurantium L. [Rutaceae; Citri aurantii fructus immaturus]*(9g)、*Scutellaria baicalensis Georgi [Lamiaceae; Scutellariae radix]*(9g)、*Pinellia ternata (Thunb.) Makino [Araceae; Pinelliae rhizoma](Thunb.) Makino*.(9g)、*Paeonia lactiflora Pall. [Paeoniaceae; Paeoniae radix alba]*(9g)、*Zingiber officinale Roscoe [Zingiberaceae; Zingiberis rhizoma]*(15g)、*Ziziphus jujuba Mill. [Rhamnaceae; Ziziphi jujubae fructus]*(12 pieces) | Prepared in accordance with the standards of the Chinese Pharmacopoeia and decocted in water. | Quality control standards for botanical drugs in compliance with the Chinese Pharmacopoeia | Yes |
| 14 | Liu WG2021 | *Bupleurum chinense DC. [Apiaceae; Bupleuri radix]*(15g)、*Rheum officinale Baill. [Polygonaceae; Rhei radix et rhizoma]*(5g)、*Citrus aurantium L. [Rutaceae; Citri aurantii fructus immaturus]*(20g)、*Scutellaria baicalensis Georgi [Lamiaceae; Scutellariae radix]*(10g)、*Pinellia ternata (Thunb.) Makino [Araceae; Pinelliae rhizoma](Thunb.) Makino*.(15g)、*Paeonia lactiflora Pall. [Paeoniaceae; Paeoniae radix alba]*(20g)、*Zingiber officinale Roscoe [Zingiberaceae; Zingiberis rhizoma]*(15g) | Prepared in accordance with the standards of the Chinese Pharmacopoeia and decocted in water. | Quality control standards for botanical drugs in compliance with the Chinese Pharmacopoeia | Yes |
| 15 | Liu YQ2015 | *Bupleurum chinense DC. [Apiaceae; Bupleuri radix]*(20g)、*Rheum officinale Baill. [Polygonaceae; Rhei radix et rhizoma]*(15g)、*Citrus aurantium L. [Rutaceae; Citri aurantii fructus immaturus]*(10g)、*Scutellaria baicalensis Georgi [Lamiaceae; Scutellariae radix]*(20g)、*Pinellia ternata (Thunb.) Makino [Araceae; Pinelliae rhizoma](Thunb.) Makino*.(10g)、*Paeonia lactiflora Pall. [Paeoniaceae; Paeoniae radix alba]*(20g) | Prepared in accordance with the standards of the Chinese Pharmacopoeia and decocted in water. | Quality control standards for botanical drugs in compliance with the Chinese Pharmacopoeia | Yes |
| 16 | Su YB2016 | *Bupleurum chinense DC. [Apiaceae; Bupleuri radix]*(15g)、*Rheum officinale Baill. [Polygonaceae; Rhei radix et rhizoma]*(15g)、*Citrus aurantium L. [Rutaceae; Citri aurantii fructus immaturus]*(10g)、*Scutellaria baicalensis Georgi [Lamiaceae; Scutellariae radix]*(10g)、*Pinellia ternata (Thunb.) Makino [Araceae; Pinelliae rhizoma](Thunb.) Makino*.(10g)、*Paeonia lactiflora Pall. [Paeoniaceae; Paeoniae radix alba]*(10g)、*Zingiber officinale Roscoe [Zingiberaceae; Zingiberis rhizoma]*(15g)、*Ziziphus jujuba Mill. [Rhamnaceae; Ziziphi jujubae fructus]*(4 pieces) | Prepared in accordance with the standards of the Chinese Pharmacopoeia and decocted in water. | Quality control standards for botanical drugs in compliance with the Chinese Pharmacopoeia | Yes |
| 17 | Sun JF2015 | *Bupleurum chinense DC. [Apiaceae; Bupleuri radix]*(15g)、*Rheum officinale Baill. [Polygonaceae; Rhei radix et rhizoma]*(15g)、*Citrus aurantium L. [Rutaceae; Citri aurantii fructus immaturus]*(12g)、*Scutellaria baicalensis Georgi [Lamiaceae; Scutellariae radix]*(10g)、*Pinellia ternata (Thunb.) Makino [Araceae; Pinelliae rhizoma](Thunb.) Makino*.(12g)、*Paeonia lactiflora Pall. [Paeoniaceae; Paeoniae radix alba]*(35g) | Prepared in accordance with the standards of the Chinese Pharmacopoeia and decocted in water. | Quality control standards for botanical drugs in compliance with the Chinese Pharmacopoeia | Yes |
| 18 | Wang EC2020 | *Bupleurum chinense DC. [Apiaceae; Bupleuri radix]*(15g)、*Rheum officinale Baill. [Polygonaceae; Rhei radix et rhizoma]*(10g)、*Citrus aurantium L. [Rutaceae; Citri aurantii fructus immaturus]*(20g)、*Scutellaria baicalensis Georgi [Lamiaceae; Scutellariae radix]*(12g)、*Pinellia ternata (Thunb.) Makino [Araceae; Pinelliae rhizoma](Thunb.) Makino*.(10g)、*Paeonia lactiflora Pall. [Paeoniaceae; Paeoniae radix alba]*(15g) | Prepared in accordance with the standards of the Chinese Pharmacopoeia and decocted in water. | Quality control standards for botanical drugs in compliance with the Chinese Pharmacopoeia | Yes |
| 19 | Wang HR2014 | *Bupleurum chinense DC. [Apiaceae; Bupleuri radix]*(15g)、*Rheum officinale Baill. [Polygonaceae; Rhei radix et rhizoma]*(6g)、*Citrus aurantium L. [Rutaceae; Citri aurantii fructus immaturus]*(9g)、*Scutellaria baicalensis Georgi [Lamiaceae; Scutellariae radix]*(9g)、*Pinellia ternata (Thunb.) Makino [Araceae; Pinelliae rhizoma](Thunb.) Makino*.(9g)、*Paeonia lactiflora Pall. [Paeoniaceae; Paeoniae radix alba]*(9g)、*Zingiber officinale Roscoe [Zingiberaceae; Zingiberis rhizoma]*(15g)、*Ziziphus jujuba Mill. [Rhamnaceae; Ziziphi jujubae fructus]*(4 pieces) | Prepared in accordance with the standards of the Chinese Pharmacopoeia and decocted in water. | Quality control standards for botanical drugs in compliance with the Chinese Pharmacopoeia | Yes |
| 20 | Wang SS2010 | *Bupleurum chinense DC. [Apiaceae; Bupleuri radix]*(12g)、*Rheum officinale Baill. [Polygonaceae; Rhei radix et rhizoma]*(10g)、*Scutellaria baicalensis Georgi [Lamiaceae; Scutellariae radix]*(10g)、*Pinellia ternata (Thunb.) Makino [Araceae; Pinelliae rhizoma](Thunb.) Makino*.(10g)、*Paeonia lactiflora Pall. [Paeoniaceae; Paeoniae radix alba]*(15g) | Prepared in accordance with the standards of the Chinese Pharmacopoeia and decocted in water. | Quality control standards for botanical drugs in compliance with the Chinese Pharmacopoeia | Yes |
| 21 | Wei YS2016 | *Bupleurum chinense DC. [Apiaceae; Bupleuri radix]*(10g)、*Rheum officinale Baill. [Polygonaceae; Rhei radix et rhizoma]*(10g)、*Citrus aurantium L. [Rutaceae; Citri aurantii fructus immaturus]*(10g)、*Scutellaria baicalensis Georgi [Lamiaceae; Scutellariae radix]*(15g)、*Pinellia ternata (Thunb.) Makino [Araceae; Pinelliae rhizoma](Thunb.) Makino*.(15g)、*Paeonia lactiflora Pall. [Paeoniaceae; Paeoniae radix alba]*(15g)、*Zingiber officinale Roscoe [Zingiberaceae; Zingiberis rhizoma]*(15g) | Prepared in accordance with the standards of the Chinese Pharmacopoeia and decocted in water. | Quality control standards for botanical drugs in compliance with the Chinese Pharmacopoeia | Yes |
| 22 | Xia CG2020 | *Bupleurum chinense DC. [Apiaceae; Bupleuri radix]*(12g)、*Rheum officinale Baill. [Polygonaceae; Rhei radix et rhizoma]*(10g)、*Citrus aurantium L. [Rutaceae; Citri aurantii fructus immaturus]*(10g)、*Scutellaria baicalensis Georgi [Lamiaceae; Scutellariae radix]*(10g)、*Pinellia ternata (Thunb.) Makino [Araceae; Pinelliae rhizoma](Thunb.) Makino*.(10g)、*Paeonia lactiflora Pall. [Paeoniaceae; Paeoniae radix alba]*(20g)、*Zingiber officinale Roscoe [Zingiberaceae; Zingiberis rhizoma]*(10g) | Prepared in accordance with the standards of the Chinese Pharmacopoeia and decocted in water. | Quality control standards for botanical drugs in compliance with the Chinese Pharmacopoeia | Yes |
| 23 | Xiong Y2021 | *Bupleurum chinense DC. [Apiaceae; Bupleuri radix]*(15g)、*Rheum officinale Baill. [Polygonaceae; Rhei radix et rhizoma]*(10g)、*Citrus aurantium L. [Rutaceae; Citri aurantii fructus immaturus]*(10g)、*Scutellaria baicalensis Georgi [Lamiaceae; Scutellariae radix]*(15g)、*Pinellia ternata (Thunb.) Makino [Araceae; Pinelliae rhizoma](Thunb.) Makino*.(10g)、*Paeonia lactiflora Pall. [Paeoniaceae; Paeoniae radix alba]*(20g) | Prepared in accordance with the standards of the Chinese Pharmacopoeia and decocted in water. | Quality control standards for botanical drugs in compliance with the Chinese Pharmacopoeia | Yes |
| 24 | Xu T2017 | *Bupleurum chinense DC. [Apiaceae; Bupleuri radix]*(15g)、*Rheum officinale Baill. [Polygonaceae; Rhei radix et rhizoma]*(15g)、*Citrus aurantium L. [Rutaceae; Citri aurantii fructus immaturus]*(10g)、*Scutellaria baicalensis Georgi [Lamiaceae; Scutellariae radix]*(10g)、*Pinellia ternata (Thunb.) Makino [Araceae; Pinelliae rhizoma](Thunb.) Makino*.(10g)、*Paeonia lactiflora Pall. [Paeoniaceae; Paeoniae radix alba]*(10g)、*Zingiber officinale Roscoe [Zingiberaceae; Zingiberis rhizoma]*(15g)、*Ziziphus jujuba Mill. [Rhamnaceae; Ziziphi jujubae fructus]*(4 pieces) | Prepared in accordance with the standards of the Chinese Pharmacopoeia and decocted in water. | Quality control standards for botanical drugs in compliance with the Chinese Pharmacopoeia | Yes |
| 25 | Yang DD2016 | *Bupleurum chinense DC. [Apiaceae; Bupleuri radix]*(15g)、*Rheum officinale Baill. [Polygonaceae; Rhei radix et rhizoma]*(10g)、*Citrus aurantium L. [Rutaceae; Citri aurantii fructus immaturus]*(10g)、*Scutellaria baicalensis Georgi [Lamiaceae; Scutellariae radix]*(10g)、*Paeonia lactiflora Pall. [Paeoniaceae; Paeoniae radix alba]*(10g)、*Zingiber officinale Roscoe [Zingiberaceae; Zingiberis rhizoma]*(10g) | Prepared in accordance with the standards of the Chinese Pharmacopoeia and decocted in water. | Quality control standards for botanical drugs in compliance with the Chinese Pharmacopoeia | Yes |
| 26 | Yu XJ2009 | *Bupleurum chinense DC. [Apiaceae; Bupleuri radix]*(15g)、*Rheum officinale Baill. [Polygonaceae; Rhei radix et rhizoma]*(10g)、*Citrus aurantium L. [Rutaceae; Citri aurantii fructus immaturus]*(15g)、*Scutellaria baicalensis Georgi [Lamiaceae; Scutellariae radix]*(10g)、*Pinellia ternata (Thunb.) Makino [Araceae; Pinelliae rhizoma](Thunb.) Makino*.(15g)、*Paeonia lactiflora Pall. [Paeoniaceae; Paeoniae radix alba]*(20g)、*Zingiber officinale Roscoe [Zingiberaceae; Zingiberis rhizoma]*(10g) | Prepared in accordance with the standards of the Chinese Pharmacopoeia and decocted in water. | Quality control standards for botanical drugs in compliance with the Chinese Pharmacopoeia | Yes |
| 27 | Zhang JY2003 | *Bupleurum chinense DC. [Apiaceae; Bupleuri radix]*(15g)、*Rheum officinale Baill. [Polygonaceae; Rhei radix et rhizoma]*(15g)、*Citrus aurantium L. [Rutaceae; Citri aurantii fructus immaturus]*(10g)、*Scutellaria baicalensis Georgi [Lamiaceae; Scutellariae radix]*(10g)、*Pinellia ternata (Thunb.) Makino [Araceae; Pinelliae rhizoma](Thunb.) Makino*.(10g)、*Paeonia lactiflora Pall. [Paeoniaceae; Paeoniae radix alba]*(10g)、*Zingiber officinale Roscoe [Zingiberaceae; Zingiberis rhizoma]*(15g)、*Ziziphus jujuba Mill. [Rhamnaceae; Ziziphi jujubae fructus]*(4 pieces) | Prepared in accordance with the standards of the Chinese Pharmacopoeia and decocted in water. | Quality control standards for botanical drugs in compliance with the Chinese Pharmacopoeia | Yes |
| 28 | Zhang N2018 | *Bupleurum chinense DC. [Apiaceae; Bupleuri radix]*(9g)、*Rheum officinale Baill. [Polygonaceae; Rhei radix et rhizoma]*(3g)、*Citrus aurantium L. [Rutaceae; Citri aurantii fructus immaturus]*(9g)、*Scutellaria baicalensis Georgi [Lamiaceae; Scutellariae radix]*(9g)、*Pinellia ternata (Thunb.) Makino [Araceae; Pinelliae rhizoma](Thunb.) Makino*.(6g)、*Paeonia lactiflora Pall. [Paeoniaceae; Paeoniae radix alba]*(9g)、*Zingiber officinale Roscoe [Zingiberaceae; Zingiberis rhizoma]*(6g)、*Ziziphus jujuba Mill. [Rhamnaceae; Ziziphi jujubae fructus]*(9g) | Prepared in accordance with the standards of the Chinese Pharmacopoeia and decocted in water. | Quality control standards for botanical drugs in compliance with the Chinese Pharmacopoeia | Yes |
| 29 | Zhou H2019 | *Bupleurum chinense DC. [Apiaceae; Bupleuri radix]*(15g)、*Rheum officinale Baill. [Polygonaceae; Rhei radix et rhizoma]*(10g)、*Citrus aurantium L. [Rutaceae; Citri aurantii fructus immaturus]*(15g)、*Scutellaria baicalensis Georgi [Lamiaceae; Scutellariae radix]*(15g)、*Pinellia ternata (Thunb.) Makino [Araceae; Pinelliae rhizoma](Thunb.) Makino*.(15g)、*Paeonia lactiflora Pall. [Paeoniaceae; Paeoniae radix alba]*(10g)、*Zingiber officinale Roscoe [Zingiberaceae; Zingiberis rhizoma]*(15g) | Prepared in accordance with the standards of the Chinese Pharmacopoeia and decocted in water. | Quality control standards for botanical drugs in compliance with the Chinese Pharmacopoeia | Yes |
| 30 | Zhou MJ2020 | *Bupleurum chinense DC. [Apiaceae; Bupleuri radix]*(15g)、*Rheum officinale Baill. [Polygonaceae; Rhei radix et rhizoma]*(10g)、*Citrus aurantium L. [Rutaceae; Citri aurantii fructus immaturus]*(15g)、*Scutellaria baicalensis Georgi [Lamiaceae; Scutellariae radix]*(15g)、*Pinellia ternata (Thunb.) Makino [Araceae; Pinelliae rhizoma](Thunb.) Makino*.(15g)、*Paeonia lactiflora Pall. [Paeoniaceae; Paeoniae radix alba]*(10g)、*Zingiber officinale Roscoe [Zingiberaceae; Zingiberis rhizoma]*(15g) | Prepared in accordance with the standards of the Chinese Pharmacopoeia and decocted in water. | Quality control standards for botanical drugs in compliance with the Chinese Pharmacopoeia | Yes |
| 31 | Zhu WB2017 | *Bupleurum chinense DC. [Apiaceae; Bupleuri radix]*(9g)、*Rheum officinale Baill. [Polygonaceae; Rhei radix et rhizoma]*(3g)、*Citrus aurantium L. [Rutaceae; Citri aurantii fructus immaturus]*(9g)、*Scutellaria baicalensis Georgi [Lamiaceae; Scutellariae radix]*(9g)、*Pinellia ternata (Thunb.) Makino [Araceae; Pinelliae rhizoma](Thunb.) Makino*.(6g)、*Paeonia lactiflora Pall. [Paeoniaceae; Paeoniae radix alba]*(9g)、*Zingiber officinale Roscoe [Zingiberaceae; Zingiberis rhizoma]*(6g)、*Ziziphus jujuba Mill. [Rhamnaceae; Ziziphi jujubae fructus]*(9g) | Prepared in accordance with the standards of the Chinese Pharmacopoeia and decocted in water. | Quality control standards for botanical drugs in compliance with the Chinese Pharmacopoeia | Yes |
| 32 | Zhuang LL2021 | *Bupleurum chinense DC. [Apiaceae; Bupleuri radix]*(15g)、*Rheum officinale Baill. [Polygonaceae; Rhei radix et rhizoma]*(10g)、*Citrus aurantium L. [Rutaceae; Citri aurantii fructus immaturus]*(15g)、*Scutellaria baicalensis Georgi [Lamiaceae; Scutellariae radix]*(15g)、*Pinellia ternata (Thunb.) Makino [Araceae; Pinelliae rhizoma](Thunb.) Makino*.(15g)、*Paeonia lactiflora Pall. [Paeoniaceae; Paeoniae radix alba]*(15g)、*Zingiber officinale Roscoe [Zingiberaceae; Zingiberis rhizoma]*(15g)、*Ziziphus jujuba Mill. [Rhamnaceae; Ziziphi jujubae fructus]*(4 pieces) | Prepared in accordance with the standards of the Chinese Pharmacopoeia and decocted in water. | Quality control standards for botanical drugs in compliance with the Chinese Pharmacopoeia | Yes |
| 33 | Zuo YZ2021 | *Bupleurum chinense DC. [Apiaceae; Bupleuri radix]*(15g)、*Rheum officinale Baill. [Polygonaceae; Rhei radix et rhizoma]*(10g)、*Citrus aurantium L. [Rutaceae; Citri aurantii fructus immaturus]*(20g)、*Scutellaria baicalensis Georgi [Lamiaceae; Scutellariae radix]*(12g)、*Pinellia ternata (Thunb.) Makino [Araceae; Pinelliae rhizoma](Thunb.) Makino*.(10g)、*Paeonia lactiflora Pall. [Paeoniaceae; Paeoniae radix alba]*(15g)、*Zingiber officinale Roscoe [Zingiberaceae; Zingiberis rhizoma]*(15g)、*Ziziphus jujuba Mill. [Rhamnaceae; Ziziphi jujubae fructus]*(4 pieces) | Prepared in accordance with the standards of the Chinese Pharmacopoeia and decocted in water. | Quality control standards for botanical drugs in compliance with the Chinese Pharmacopoeia | Yes |

## Table 2 Characteristics and main findings of mechanism studies

| Study | Animal models | Intervention | Disease | Action mechanism |
| --- | --- | --- | --- | --- |
| Bi SJ2023^[1]^ | Cholecystitis guinea pig model | Da Chaihu decoction | Cholecystitis | MMP9↑, JAK2↑, MAP2K1↑, NR3C1↓ |
| Zhang T2021^[2]^ | Calculous cholecystitis guinea pig model | Da Chaihu decoction | Acute Calculous cholecystitis | TNF-α↓, CCK↑, SOD↑ |
| Ma YX2023^[3]^ | Acute Calculous cholecystitis guinea pig model | Da Chaihu decoction | acute cholecystitis | TNF-α↓,IL-1β↓ , Cav-3↓ |
| Ping J2005^[4]^ | Acute cholecystitis rabbit model | Da Chaihu decoction | acute cholecystitis | Improvement of blood circulation |

[1] Bi S, Liu Y, Lv T, et al. Preliminary exploration of method for screening efficacy markers compatibility in TCM prescriptions based on Q-markers: Anti-inflammatory activity of Dachaihu decoction as an example.J Ethnopharmacol. 2023;312:116539. doi:10.1016/j.jep.2023.116539

[2]Zhang T. Study on the mechanism of Dachaihu decoction in the treatment of calculous cholecystitis based on network pharmacology [Master Dissertation]. Beijing, China: Beijing University of Chinese Medicine, 2021.DOI:10.26973/d.cnki.gbjzu.2021.000217.

[3]Ma YX, Zhou CY, Yang CC, et al. Basic study on the mechanism of Da chaihu decoction in the treatment of Calculous cholecystitis [J]. Modern journal of Integrated traditional Chinese and Western medicine,2023,32(15):2082-2086+2167. Doi: 10.3969/j.issn.1008-8849.2023.15.007

[4] Ping J. Research on mechanism of Da Chai Hu Tang on treating Acute cholecystitis [Master Dissertation]. Shandong, China: Shandong University of Traditional Chinese Medicine,2005.

**Supplementary Material 2**

**Table 1**

Search strategy for PubMed.

| Search query |
| --- |
| **#1** daisaikoto[MeSH Terms]  **#2** daisaikoto[Title/Abstract] OR Dai-saiko-to[Title/Abstract] OR Da-Chai-Hu-Tang[Title/Abstract] OR da chai hu tang[Title/Abstract] OR dachaihu tang[Title/Abstract] OR dachaihu-tang[Title/Abstract] OR dachaihu decoction[Title/Abstract] OR Dachaihu decoction[Title/Abstract] OR Da chaihu decoction[Title/Abstract] OR dai saiko to[Title/Abstract] OR dai-saiko-to[Title/Abstract] OR daisaiko-to[Title/Abstract] OR major bupleurum decoction[Title/Abstract] OR tj 8[Title/Abstract]  **#3** #1 OR #2  **#4** Cholecystitis, Acute[MeSH Terms]  **#5** Cholecystitis, Acute[Title/Abstract] OR Acute Cholecystitis[Title/Abstract]  **#6** #4 OR #5  **#7** #3 AND #6 |
| **Results 1** |

**Table 2**

Search strategy for Embase.

| Search query |
| --- |
| **#1** 'daisaikoto'/exp OR 'daisaikoto':ab,ti OR 'da chai hu tang':ab,ti OR 'da-chai-hu-tang':ab,ti OR 'dachaihu tang':ab,ti OR 'dachaihu-tang':ab,ti OR 'dachaihu decoction':ab,ti OR 'dai saiko to':ab,ti OR 'dai-saiko-to':ab,ti OR 'daisaiko to':ab,ti OR 'daisaiko-to':ab,ti OR 'daisaikotoh':ab,ti OR 'major bupleurum decoction':ab,ti OR 'tj 8':ab,ti  **#2** 'acute cholecystitis'/exp OR 'acute cholecystitis':ab,ti OR 'cholecystitis, acute':ab,ti  **#3** #1 AND #2 |
| **Results 1** |

**Table 3**

Search strategy for WOS.

| Search query |
| --- |
| **#1** TS=("daisaikoto" or "da chai hu tang" or "da-chai-hu-tang" or"dachaihu tang" or "dachaihu-tang" or "dachaihu decoction" or "dai saiko to" or "dai-saiko-to" or "daisaiko to" or "daisaiko-to" "daisaikotoh" or "major bupleurum decoction" or "tj 8")  **#2** TS=("acute cholecystitis" or "acute cholecystitis" or "cholecystitis, acute")  **#3** #1 and #2 |
| **Results 0** |

**Table 4**

Search strategy for Cochrane Library.

| Search query |
| --- |
| **#1** (daisaikoto):ti,ab,kw OR (da chai hu tang):ti,ab,kw OR (da-chai-hu-tang):ti,ab,kw OR (dachaihu tang):ti,ab,kw OR (dachaihu-tang):ti,ab,kw  **#2** (dachaihu decoction):ti,ab,kw OR (dai saiko to):ti,ab,kw OR (dai-saiko-to):ti,ab,kw OR (daisaiko to):ti,ab,kw OR (dai-saiko-to):ti,ab,kw  **#3** (daisaikotoh):ti,ab,kw OR (major bupleurum decoction):ti,ab,kw OR (tj 8):ti,ab,kw  **#4** #1 OR #2 OR #3  **#5** MeSH descriptor: [Cholecystitis, Acute] explode all trees  **#6** (Cholecystitis, Acute):ti,ab,kw OR (Acute Cholecystitis):ti,ab,kw  **#7** #5 OR #6  **#8** #4 AND #7 |
| **Results 13** |
| **Table 5**  Search strategy for CNKI. |
| **#1** (SU=(大柴胡汤) OR SU=(大柴胡颗粒) OR SU=(大柴胡汤加味) OR SU=(大柴胡汤加减) OR SU=(大柴胡汤加减方) TKA=(大柴胡汤) OR TKA=(大柴胡颗粒) OR TKA=(大柴胡汤加味) OR TKA=(大柴胡汤加减) OR TKA=(大柴胡汤加减方))  **#2** (SU=(急性胆囊炎) OR TKA=(急性胆囊炎))  **#3** #1 AND #2 |
| **Results 121** |

| **Table 6**  Search strategy for Wanfang. |
| --- |
| **#1** (主题:(大柴胡汤 or 大柴胡颗粒 or 大柴胡汤加味 or 大柴胡汤加减 or 大柴胡汤加减方))  **#2** (主题:(急性胆囊炎))  **#3** #1 and #2 |
| **Results 153** |

**Table 7**

Search strategy for VIP.

| Search query |
| --- |
| **#1** ((M=(大柴胡汤 OR 大柴胡颗粒 OR 大柴胡汤加味 OR 大柴胡汤加减 OR 大柴胡汤加减方) OR R=(大柴胡汤 OR 大柴胡颗粒 OR 大柴胡汤加味 OR 大柴胡汤加减 OR 大柴胡汤加减方)))  **#2** ((M=(急性胆囊炎)) OR (R=(急性胆囊炎)))  **#3** #1 and #2 |
| **Results 163** |

**Table 8**

Search strategy for SinoMed.

| Search query |
| --- |
| **#1** ("大柴胡汤"[常用字段:智能] OR "大柴胡颗粒"[常用字段:智能] OR "大柴胡汤加味"[常用字段:智能] OR "大柴胡汤加减"[常用字段:智能] OR "大柴胡汤加减方"[常用字段:智能])  **#2** ("急性胆囊炎"[常用字段:智能])  **#3** #1 AND #2 |
| **Results 97** |

**Table 9**

Search strategy for ICTRP.

| Search query |
| --- |
| **#1** Title: Acute Cholecystitis  **#2** daisaikoto OR Dai-saiko-to OR Da-Chai-Hu-Tang OR da chai hu tang OR dachaihu tang OR dachaihu-tang OR dachaihu decoction OR dai saiko to OR dai-saiko-to OR daisaiko-to OR major bupleurum decoction OR tj 8  **#3** (#1) AND (#2) |
| **Results 0** |

**Table 10**

Search strategy for ClinicalTrials.gov.

| Search query |
| --- |
| **#1** Condition or disease: Acute Cholecystitis  **#2** Intervention/treatment: daisaikoto OR Dai-saiko-to OR Da-Chai-Hu-Tang OR da chai hu tang OR dachaihu tang OR dachaihu-tang OR dachaihu decoction OR dai saiko to OR dai-saiko-to OR daisaiko-to OR major bupleurum decoction OR tj 8  **#3** #1 AND #2 |
| **Results 0** |

**Table 11**

Search strategy for ChiCTR.

| Search query |
| --- |
| #1 研究疾病名称: 急性胆囊炎  #2 干预措施：大柴胡汤  #3 #1 AND #2 |
| **Results 0** |

**Supplementary Material 3**

**
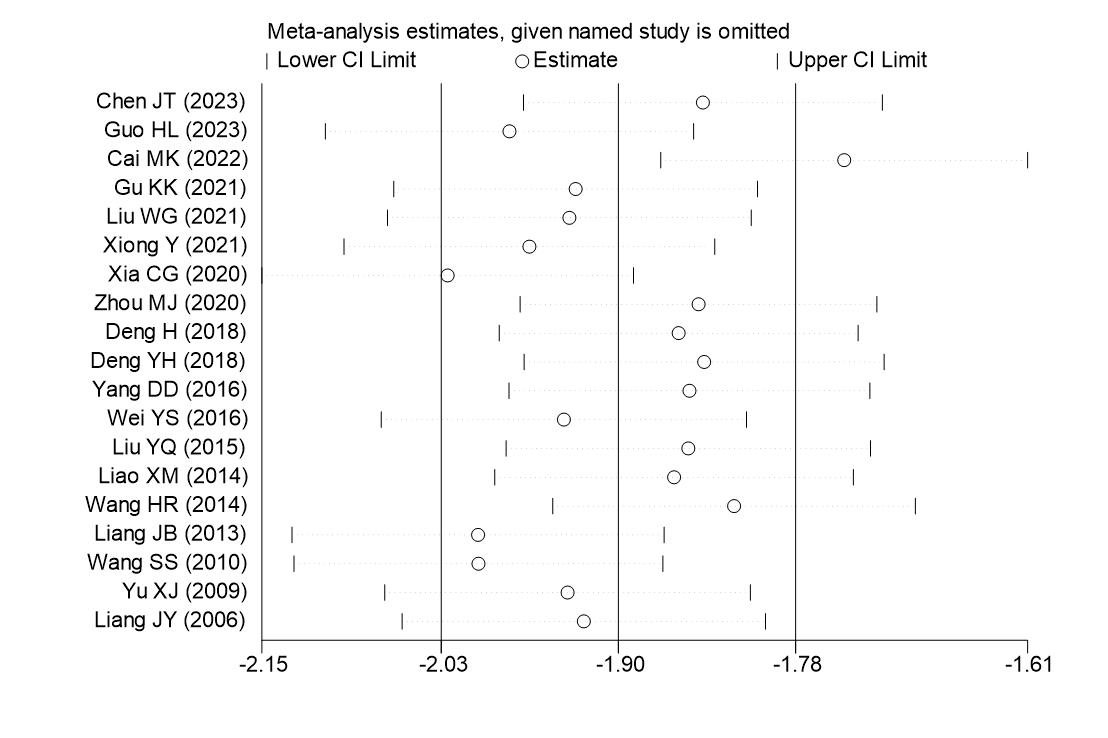
**

(1) Time for abdominal pain to disappear

**
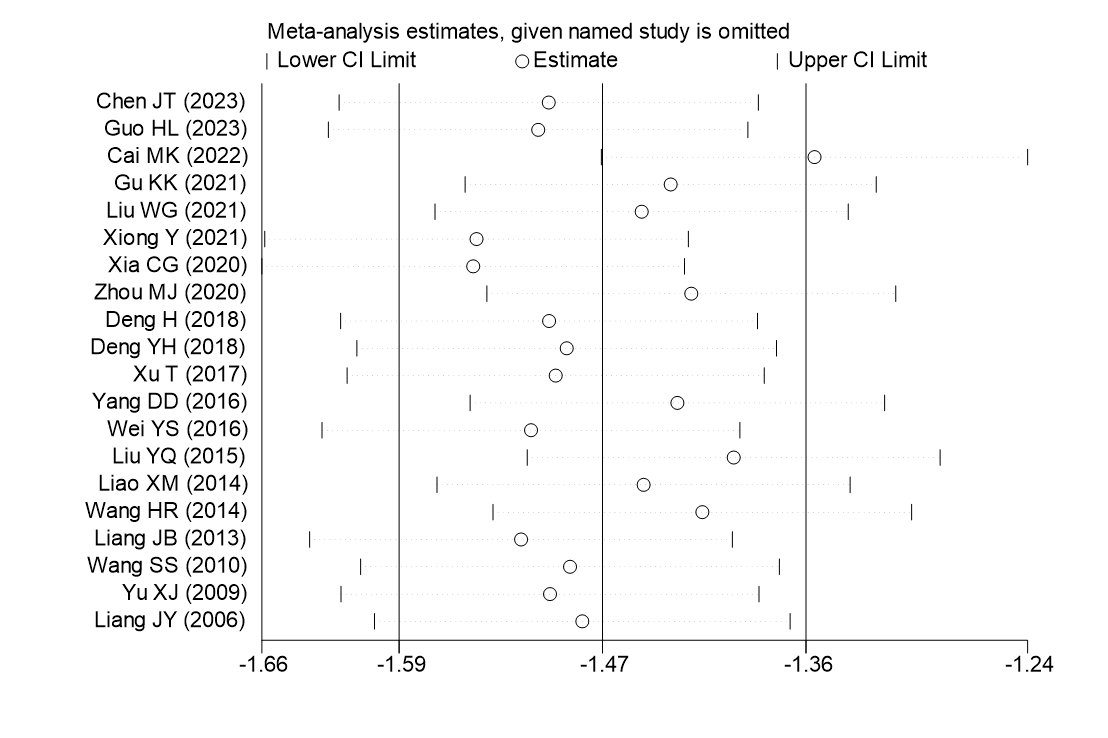
**

(2) Time for fever to disappear

**
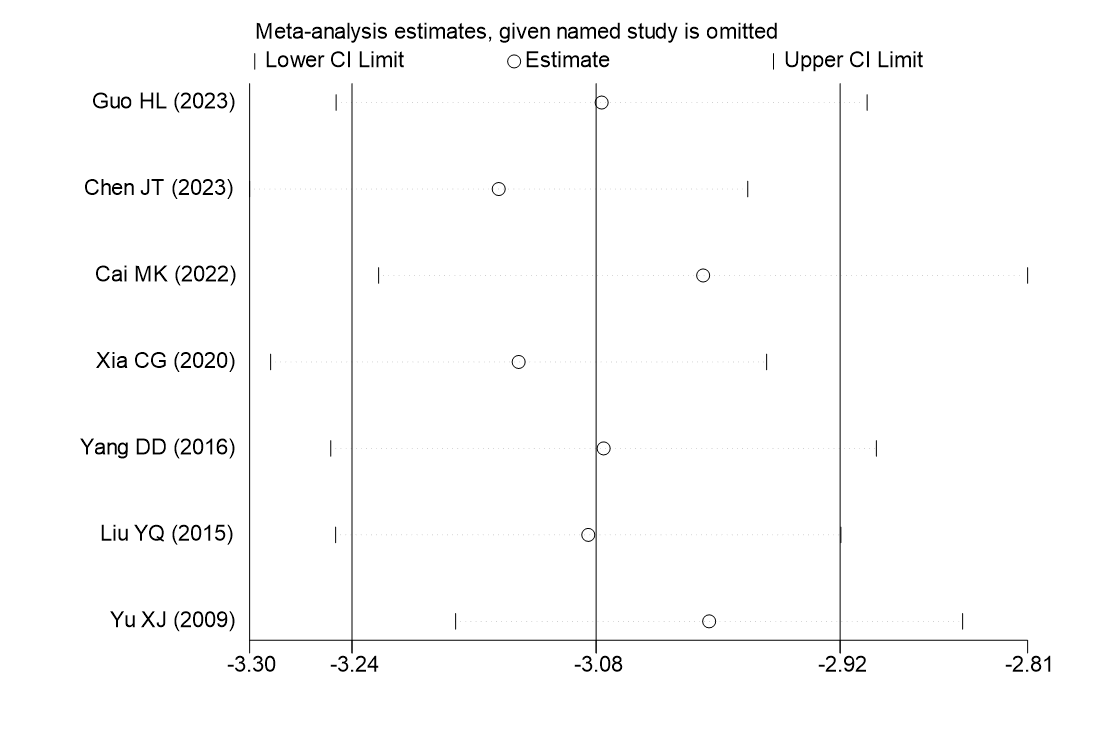
**

(3) Time for white blood cell counts to return to normal

**
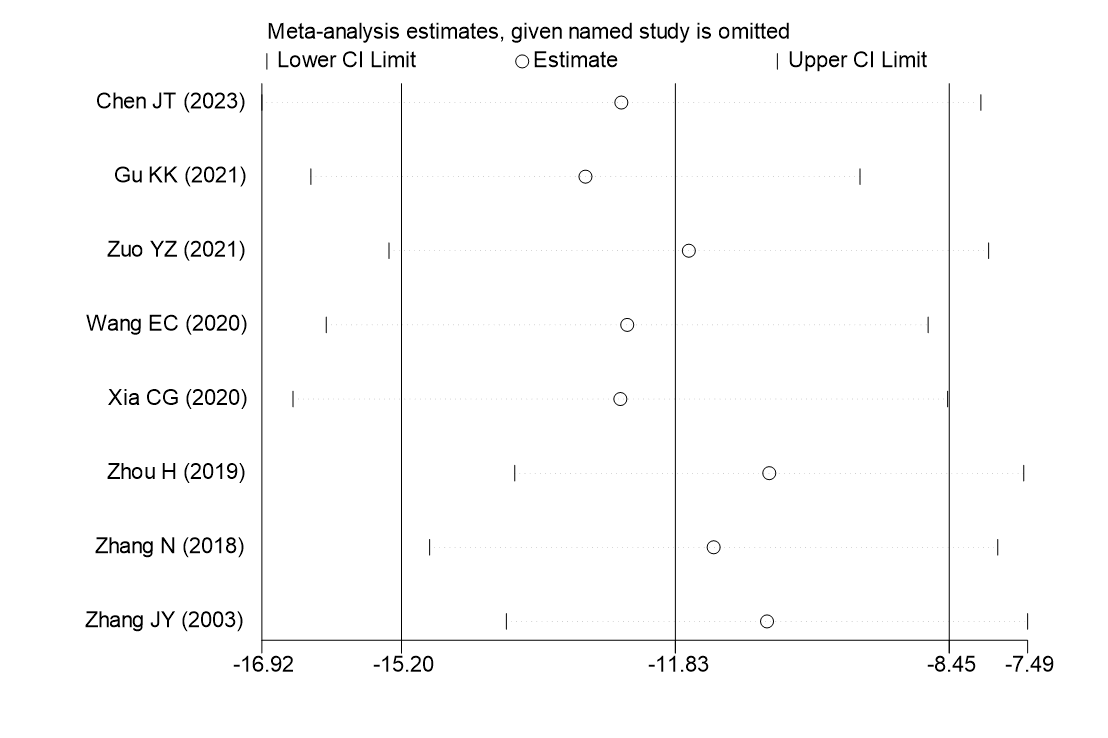
**

(4) ALT
